# Staphylococci phages display vast genomic diversity and evolutionary relationships

**DOI:** 10.1186/s12864-019-5647-8

**Published:** 2019-05-09

**Authors:** Hugo Oliveira, Marta Sampaio, Luís D. R. Melo, Oscar Dias, Welkin H. Pope, Graham F. Hatfull, Joana Azeredo

**Affiliations:** 1 CEB – Centre of Biological Engineering, University of Minho, Braga, Portugal; 2 Department of Biological Sciences, University of Pittsburgh, Pittsburgh, PA USA

**Keywords:** *Staphylococcus*, Bacteriophages, Genomes, Clusters, Phams, Endolysin

## Abstract

**Background:**

Bacteriophages are the most abundant and diverse entities in the biosphere, and this diversity is driven by constant predator–prey evolutionary dynamics and horizontal gene transfer. Phage genome sequences are under-sampled and therefore present an untapped and uncharacterized source of genetic diversity, typically characterized by highly mosaic genomes and no universal genes. To better understand the diversity and relationships among phages infecting human pathogens, we have analysed the complete genome sequences of 205 phages of *Staphylococcus* sp.

**Results:**

These are predicted to encode 20,579 proteins, which can be sorted into 2139 phamilies (phams) of related sequences; 745 of these are orphams and possess only a single gene. Based on shared gene content, these phages were grouped into four clusters (A, B, C and D), 27 subclusters (A1-A2, B1-B17, C1-C6 and D1-D2) and one singleton. However, the genomes have mosaic architectures and individual genes with common ancestors are positioned in distinct genomic contexts in different clusters. The staphylococcal Cluster B *siphoviridae* are predicted to be temperate, and the integration cassettes are often closely-linked to genes implicated in bacterial virulence determinants. There are four unusual endolysin organization strategies found in *Staphylococcus* phage genomes, with endolysins predicted to be encoded as single genes, two genes spliced, two genes adjacent and as a single gene with inter-lytic-domain secondary translational start site. Comparison of the endolysins reveals multi-domain modularity, with conservation of the SH3 cell wall binding domain.

**Conclusions:**

This study provides a high-resolution view of staphylococcal viral genetic diversity, and insights into their gene flux patterns within and across different phage groups (cluster and subclusters) providing insights into their evolution.

**Electronic supplementary material:**

The online version of this article (10.1186/s12864-019-5647-8) contains supplementary material, which is available to authorized users.

## Background

Bacteriophages (phages) – viruses of bacteria – are ubiquitous, and are the most populous (over 10^31^) and diverse of all biological entities [1, 2]. Phage predation affects not only the microbial balance [3, 4], but also food webs [5], biogeochemical cycles [6] and human diseases [7]. Phages are able to kill 50% of the bacteria produced every 48 h, playing a major role in microbial ecology and in the evolution of bacterial genomic structures through horizontal gene transfer (HGT), including virulence factors [8].

Up to January 2019, there have been 5595 complete *Caudovirales* genome sequences recorded in the RefSeq database at GenBank. The *Caudovirales* (tailed phages with dsDNA), are the most commonly isolated viruses. Phages of phylogenetically distant hosts, and often from the same host, typically share little or no DNA sequence similarity, and no universal genes [9], confounding their taxonomic classification. While nucleotide sequence-based methods such as pairwise genome alignment using BLASTN, average nucleotide identity (ANI), or dot plot analysis are useful for studying closely-related phages, analyses using shared gene content based on protein sequence similarity enlighten more distant relationships, and illustrate the diversity continuum in viral sequence space [10, 11]. These studies were undertaken for phages of *Mycobacterium* sp. (*n* = 627) [12], Enterobacteria (*n* = 337) [13], *Bacillus* sp. (*n* = 93) [14], *Gordonia* sp. (*n* = 79) [10] and *Arthrobacter* sp. (*n* = 46) hosts [15]. *Mycobacterium* phages represent the largest group of phages infecting a single host, *Mycobacterium smegmatis* mc^2^155; and early studies highlighted their high genetic diversity and genome mosaicism [16, 17]. A recent study analysed over 700 genomes of Actinobacteria phages that could be sorted into 30 distinct phage clusters [10]. The Enterobacteria phages, isolated by several investigators on multiple hosts, were sorted into 56 clusters; phage of *Bacillus* sp.*, Gordonia* sp. and *Arthrobacter* sp., were likewise sorted into related groups [10, 14, 15]. Although these surveys included hosts of different taxonomic levels, there is an evident genetic phage diversity that often includes genomes with mosaic architectures and genes of unknown function which lack homology [18].

A previous study compared the genomes of 85 *Staphylococcus* phages, mostly isolated from *S. aureus* host, and grouped them into three classes (Class I, Class II and Class III) based on their genome size, gene order, and nucleotide and protein sequences [19]. Here, we have extended the comparative genomic analysis to 205 phages infecting several species of staphylococci. We comparatively analyzed the genomes at the nucleotide and proteomic level and used a 35% shared gene content cut-off to place phages solely in one cluster. These phages, which were isolated at various times and from different environments, provide a high-resolution view of the genetic diversity among all members infecting these clinical relevant pathogens.

## Results

### Staphylococcal phages can be grouped in four clusters, 27 subclusters and one singleton

To determine the relationship of staphylococci phages, all complete genomes sequences deposited at GenBank as of October 2018 were retrieved and analysed using ANI, shared gene content and gene content dissimilarity metrics as recently described [10]. BLASTN and average nucleotide identity to identify whole phage genomes and genome regions with nucleotide sequence similarity and Phamerator to generate protein phamilies (phams) for calculating pairwise shared gene content and genome architecture. The dataset includes 205 genomes ranging from 16.8 kb (phage 44AHJD) to 151.6 kb (phage vB_SauM_0414_108) in size, coding between 20 to 249 predicted genes, and isolated from eleven different hosts, including nine coagulase-negative and three coagulase-positive or variable species (Additional file 1).

**Fig. 1:**
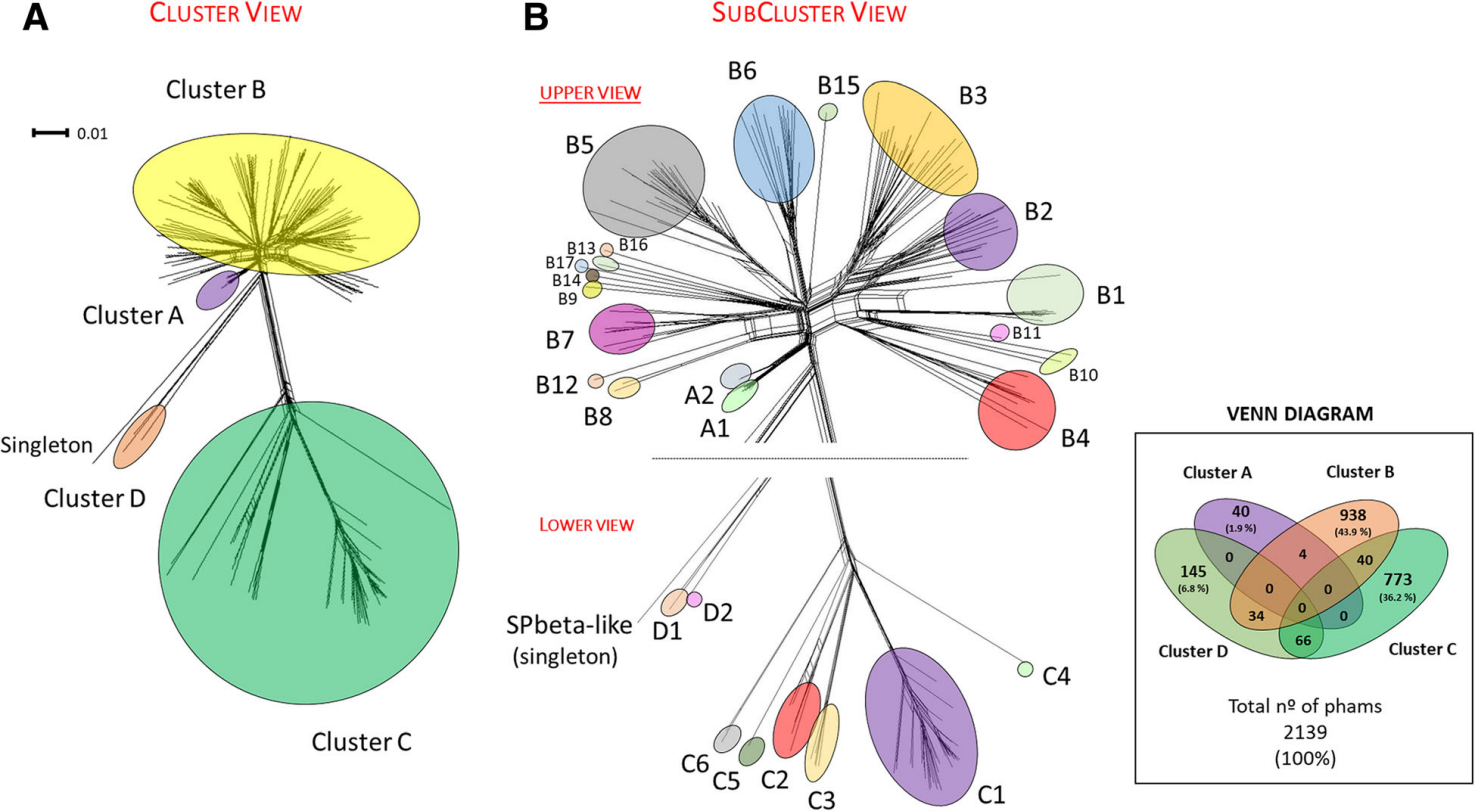
Diversity of staphylococcal phage genomes. **a**) Splitstree 3D representation into 2D space of the 205 staphylococcal phages illustrating shared phams generated from a total of 20,579 predicted genes. A total of 2139 phams (a group of genes with related sequences) of which 745 orphams (a single gene without related sequences) were identified. **b**) The assignment of A) clusters and B) subclusters are shown in coloured circles. The scale bar indicates 0.01 substitution. The spectrum of diversity reveals four clusters and 31 subclusters (A1-A2, B1-B21, C1-C6 and D1-D2) and one singleton (phage SPbeta-like). A Venn diagram was also included to visualize the amount of proteins allocated and shared across each cluster. Common phams among different clusters that are represented by intersections of the circles. There is no universal pham in staphylococci phage genomes

Comparative analysis of all 205 staphylococcal phage genomes identified 20,579 predicted proteins, which were sorted into 2139 phamilies (phams) of related sequences, 745 of which possess only a single sequence (orphams) (Additional file 2). Based on average shared gene content as determined by pham membership, these phages can be grouped into four clusters (A-D), 27 subclusters (A1-A2, B1-B17, C1-C6 and D1-D2) and one singleton (with no close relatives) (Fig. 1). A threshold value of 35% average pairwise shared gene content was used to cluster genomes, as described for *Gordonia* and *Mycobacterium* phages [10, 12]. These groupings are supported by pairwise ANI values (Additional file 3) and gene content similarity (Additional file 4). Cluster members exhibit similar virion morphology and genometrics (size, number of ORF and GC content) (Additional file 1). To further analyse relationships, we defined conserved (phams found in all phages), accessory (phams present in at least three phages) and unique (orphams, present in only one phage) phams amongst members of each cluster/subclusters, providing further insights into specific gene pattern exchanges (Additional file 5). Specific examples are provided below.

#### Cluster A

The sixteen Cluster A staphylococci phages are morphologically podoviral and can be divided into two subclusters (A1, A2). Cluster A phages are an extremely well-conserved group with respect to nucleotide and amino acid homology, morphology, lytic lifestyle, genome size (16–18 kb), GC content (27–29%), and predicted number of genes (20 to 22) (Additional file 1). The genomes are organized into left and right arms, with rightwards- and leftwards-transcription in the left and right arms, respectively (Additional files 6, 7). Interestingly, the DNA packaging and DNA polymerase genes are located near the start of the left genome terminus, with the other structural protein genes located in the right arm [20]. Subcluster A1 has 14 phages (e.g. BP39, GRCS) that share substantial ANI (> 86%) and gene content (> 82%) (Additional file 6), but differ in arrangements of the tail fiber genes (44AHJD, SLPW and 66). Subcluster A2 includes two phages (St134 and Andhra), that infect *S. epidermidis* (Additional file 7). These phages have high ANI (92%) and shared gene content (98%) values. Subcluster A1 and A2 phages vary in a tail endopeptidase gene upstream of the DNA encapsulation protein. Overall, the high number of conserved phams (17 to 20) and limited number of accessory phams (1 to 5) or unique phams (1 to 2) reflects the genomic homogeneity of Cluster A phages (Additional file 5). About 60% of genes have predicted functions related to DNA replication (DNA binding, DNA polymerase), virion morphology (DNA packaging, tail fiber, collar and major capsid) or cell lysis (holin and endolysin) (Additional file 2).

#### Cluster B

Cluster B is the largest and most diverse cluster, with 132 phage isolates from multiple different hosts (*S. aureus, S. epidermidis, S. pseudintermedius, S. sciuri*, *S. haemolyticus*, *S. saprophyticus*, *S. capitis* and *S. warneri*). Most are predicted to be temperate and the genome sizes vary from 39.6 to 47.8 kb with 42–79 predicted protein-encoding genes. The genomes are organized into a rightwards-transcribed left arm containing structural genes and the lysis cassette, a central leftwards-transcribed integration cassette, and a rightwards-transcribed right arm coding for many small proteins of unknown functions (Additional files 8, 9, 10, 11, 12, 13, 14, 15, 16, 17, 18, 19, 20, 21, 22, 23, 24). Cluster B phages are divided into 17 subclusters based on manual inspection of gene content similarity, genome pairwise comparisons, and ANI values (Additional files 8, 9, 10, 11, 12, 13, 14, 15, 16, 17, 18, 19, 20, 21, 22, 23, 24, Additional files 3, 4). The larger subclusters are B1 (*n* = 7), B2 (*n* = 19), B3 (*n* = 26), B4 (*n* = 9), B5 (n = 26), B6 (*n* = 18) and B7 (*n* = 12) and have phages with collinear genomes (Additional file 8, 9, 10, 11, 12, 13, 14). While subclusters B1-B2 and B3-B7 were exclusively isolated from *S. pseudintermedius* or *S. aureus* hosts, B4 is unusual in having phages isolated from *S. aureus, S. haemolyticus* and *S. epidermidis* (Additional file 1). The remaining B8-B17 subclusters each contain only three or fewer members, mostly isolated from rarer coagulase-negative hosts, such as *S. sciuri*, *S warneri*, *S saprophyticus S. haemolyticus* and *S. hominis*. Although they have similar genome organizations to other Cluster B phages, fewer than 42% of their genes are shared with them (Additional file 15, 16, 17, 18, 19, 20, 21, 22, 23, 24).

Cluster B phages are predicted to be temperate, and encode predicted integrase and repressor genes; prophage establishment had been demonstrated for phages phiPV83, phiNM1, phiNM2, phiNM4, vB_SepiS-phiIPLA5, vB_SepiS-phiIPLA7, 11, 42E, phi12 and phi13 [21–24]. Generally, in Cluster B genomes, about 40–50% of the predicted genes are functionally annotated with roles of DNA packaging, virion structure, cell lysis, lysogeny, or DNA replication. Overall, the spectrum of diversity of this large Cluster B is high and although all members are related through gene content similarity to at least one of the phages (> 35%), some viruses (e.g. IME1367_01, IME-SA4, phiRS7, StB20, StB20-like) have lower pairwise shared gene content (< 35%). Subcluster B1 is by far the most conserved B subcluster, with members sharing 46 conserved phams, while subcluster B2 and B4 are the most heterogeneous groups with only ten or fewer conserved phams (Additional file 5). Less than 50% protein-encoding genes have known functions in the Cluster B phages.

#### Cluster C

The 53 Cluster C phages are morphologically members of *Myoviridae*, with genome sizes ranging from 127.2 kb to 151.6 kb coding for 164–249 predicted proteins. Cluster C can be divided into six subclusters. Cluster C1 phage genomes are characterized by direct terminal repeats, base pair 1 of these genomes is selected to be the first base of the repeat; for other Cluster C phages base pair 1 is identified as the first base of the terminase gene (as per convention). Most genes are transcribed-rightwards, with the rightmost 20 kb transcribed leftwards (Additional files 25, 26, 27, 28, 29, 30). While the variation in predicted gene content is due in part to small insertion/deletions, some (10%) arise from inconsistencies in the annotations.

Subcluster C1 (*n* = 37) is the most numerous Cluster C subcluster comprised of *S. aureus* infecting phages (e.g. K and P108) (Additional file 25), and are well-conserved with ANIs > 71% and shared gene content > 72% (Additional files 3, 4). Cluster C1 phages have direct terminal repeats of ~ 8 kb, suggesting a common dsDNA packaging mechanism (Additional file 1). This subcluster is composed of phages described to have broad-host range (e.g. K) and with therapeutic potential [25].

Subcluster C2 (*n* = 6) has closely related *S. aureus*-infecting phages (Stau2, StAP1, vB_SauM_Remus, vB_SauM_Romulus, SA11 and qdsa001), with high ANI (> 95%) and shared gene content (> 77%) values (Additional file 26). They encode between 164 to 199 genes; Stau2 and Sa11 are the only members known to encode RNA ligase. The remaining phages are distributed between subclusters C3 (*n* = 5, phiIPLA-C1C, phiIBB-SEP1, Terranova, Quidividi and Twillingate), C4 (Twort), C5 (vB_SscM-1 and vB_SscM-2) and C6 (phiSA_BS1 and phiSA_BS2), respectively (Additional files 27, 28, 29, 30). All members of subclusters C3, C4 and C5 share fewer than 60% of their genes with other phages of Cluster C; these phages, such as Twort, are known to infect rare serotypes of host species that share limited nucleotide identity to *S. aureus*. Overall, all Cluster C phages have a relatively high number of shared phams (Additional file 5), but fewer than 40% of their genes have predicted functions.

#### Cluster D

Cluster D is comprised of three lytic *Siphoviridae*, 6ec, vB_SepS_SEP9 and vB_StaM_SA2, with genome sizes ranging from ∼89–93 kb, coding for 129–142 predicted proteins. The genomes have defined cohesive termini with 10 base 3′ single stranded DNA extensions (Additional file 1) [26]. The left arms are rightwards-transcribed and code for virion proteins, cell lysis functions (holin and endolysin) and predicted general recombinases (Additional files 31, 32). The right arms are leftwards-transcribed, with a leftwards-transcribed five kb insertion near the right genome end (Additional files 31, 32). The right arm contains genes with predicted functions in DNA replication (e.g. DNA polymerase) and DNA metabolism (e.g. ribonucleotide reductase) genes. The two short rightmost operons code for small proteins of unknown function. Cluster D phages do not have predicted lysogeny functions, although they code for a tyrosine recombinase in the left arm (pham 1333); a similar arrangement has been identified in lytic *Gordonia* phages [10]. It is unclear what specific role these recombinases play. Morphologically, phages 6ec and SEP9 have very long flexible tails (> 300 nm), twice as long as those of Cluster B phages [26, 27]. We also note that phage vB_SepS_SEP9 has relatively high G + C content of 45.8, 10% higher than the other staphylococcal phages (Additional file 1). This may reflect either a broader host range than other staphylococcal phages, or be a consequence of its recent evolutionary history [27].

Cluster D is subdivided into two subclusters based on ANI. Subcluster D1 has two members (6ec, vB_SepS_SEP9) with high ANI (78%) and shared gene content (77%) values and are organized collinearly (Additional file 31). Subcluster D2 has a single member (vB_StaM_SA2), which shares 45% or fewer genes with the subcluster D1 phages (Additional file 32). Although not yet examined by electron microscopy, vB_StaM_SA2 is predicted to have a similarly long noncontractile tail found in subcluster D1 members due to the similarity between the tail proteins, particularly the tape measure proteins (see pham 814 of Additional file 2). Cluster D phages have functions assigned only to about 35% of the predicted genes.

#### Phage SPbeta-like

The singleton phage SPbeta-like is a siphovirus sharing fewer than 10% of its genes with other staphylococcal phages (Additional file 33). SPbeta-like has a genome of 127,726 bp and encodes 177 genes organized into three major operons, of which only 30% have predicted functions; these include virion proteins (e.g. tape measures protein), cell lysis (holin and endolysin), DNA replication (e.g. DNA polymerase and helicase), and three predicted recombinases (phams 139, 415, 1023). Similarly to Cluster D phages, SPbeta-like lacks genes associated with stable maintenance of lysogeny.

### Gene content reflects the diversity of *Staphylococcus* phages

**Fig. 2:**
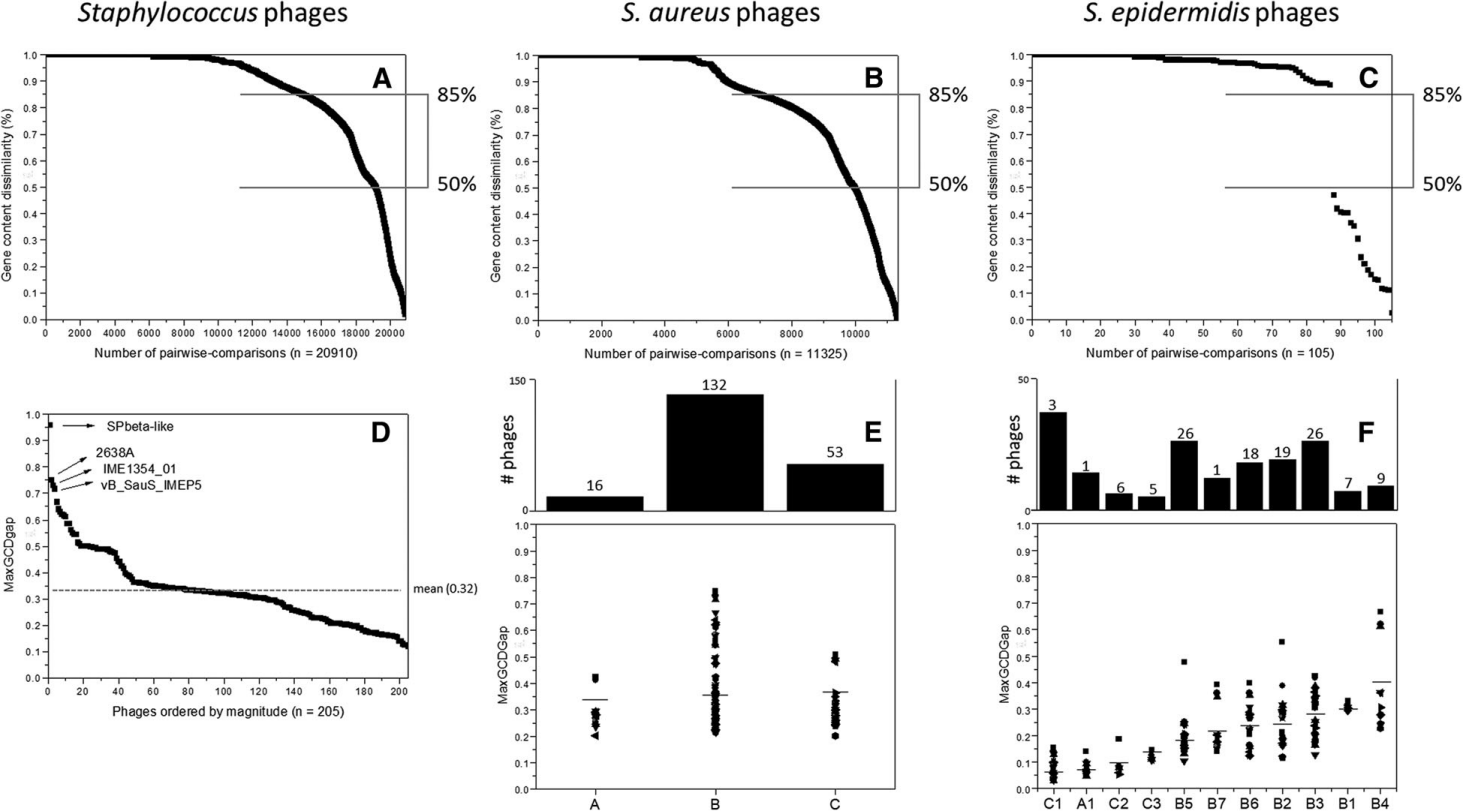
Phage relationship under gene content dissimilarity index. GCD scores given by each pairwise comparison for **a**) all staphylococcal, **b**) *S. aureus* phage genomes or **c**) *S. epidermidis* phage genomes (where GCD = 1 meaning 100% dissimilar, GCD = 0 meaning 100% similar). **d** MaxGCDGap relationships for all staphylococcal phages ordered by median (where higher MaxGCDGap mean most diverse and lower MaxGCDGap mean less diverse, relative to the groups analysed). MaxGCDGap relationships for **e**) cluster of phages (**a** to **d**) or for **f**) subclusters of phages (A1-A2, B1-B21, C1-C6, D1-D2) and the singletons, where each data point represents a single phage genome. Horizontal lines show the MaxGCDGap mean per cluster and subclusters. Cluster and subclusters with less than five members were omitted from the analysis in **e** and **f**

To further assess diversity of *Staphylococcus* phages and clusters, we calculated pairwise gene content dissimilarity (GCD) and maximum GCD gap distance (MaxGCDGap) metrics (Fig. 2a-f), as described previously [10, 11]. The GCD metric ranges from 1 (no shared 0 genes) to 0 (all genes are shared). We generated three datasets, the first including *Staphylococcus* sp. phages (*n* = 205), the second with only those isolated on *S. aureus* (*n* = 162), and the third including *S. epidermidis* phages (n = 16) (Fig. 2a-c). Of 20,910 staphylococcal phage pairwise comparisons, the majority (78%) share 20% or fewer genes (GCD > 0.8), (Fig. 2a); likewise, of 11,325 *S. aureus* phage pairwise comparisons, 71% had 20% or fewer shared genes (GCD > 0.8) (Fig. 2b). However, within the 105 *S. epidermidis* phage pairwise comparisons, 83% had 20% or fewer shared genes (GCD > 0.8), (Fig. 2c). *Staphylococcus* sp. and *S. aureus*-infecting phages exhibited a number of pairwise comparisons (∼25%) that yielded GCD values between 0.85 and 0.50, reflecting between 15 and 50% shared genes, respectively. None of the *S. epidermidis* phage pairwise comparisons were found in this range, indicating that the *S. epidermidis* phages primarily shared phams with closely related phages, and not with unrelated phages.

Rank ordered GCD pairwise comparisons illustrate the continuum of diversity found in any particular set of phages with sufficient members; the largest difference between two adjacent points is termed MaxGCDGap. Phages in datasets with a large MaxGCDGap exhibit cluster isolation, with fewer phages sharing phams with non-cluster members. MaxGCDGap can range from near 0 (indicating small gene content discontinuities, all phages are closely related) to 1 (indicating large gene content discontinuities, no phages are closely related). Although this metric is dependent on the dataset size and composition, the spectrum of genetic diversity can be further resolved with additional genomes [10]. With the exception of SPbeta-like, MaxGCDGap values show an almost uninterrupted spectrum from 0.75 to 0.12, with a mean value of 0.33 (Fig. 2d), the singleton SPbeta-like has a much higher MaxGCDGap value of 0.96, as expected. We also plotted MaxGCDGap values ordered by magnitude per cluster and per subcluster (Fig. 2e-f), showing a broad range of values, reflecting the spectrum of diversity in the entire phage genome set. We noted a lower variability of MaxGCDGap in clusters A and C, indicative of that they are well-conserved groups, in comparison with Cluster B (and in particular subcluster B4), that possess broader range and higher MaxGCDGap values reflecting a greater diversity. Similar observations of different levels of gene content discontinuities have been described previously, with *Propionibacterium* or *Arthrobacter* phages and Mycobacteria or *Synechococcus* phages, as examples of good and poorly conserved groups, respectively [10].

### Staphylococci phages display multiple integration systems

**Fig. 3:**
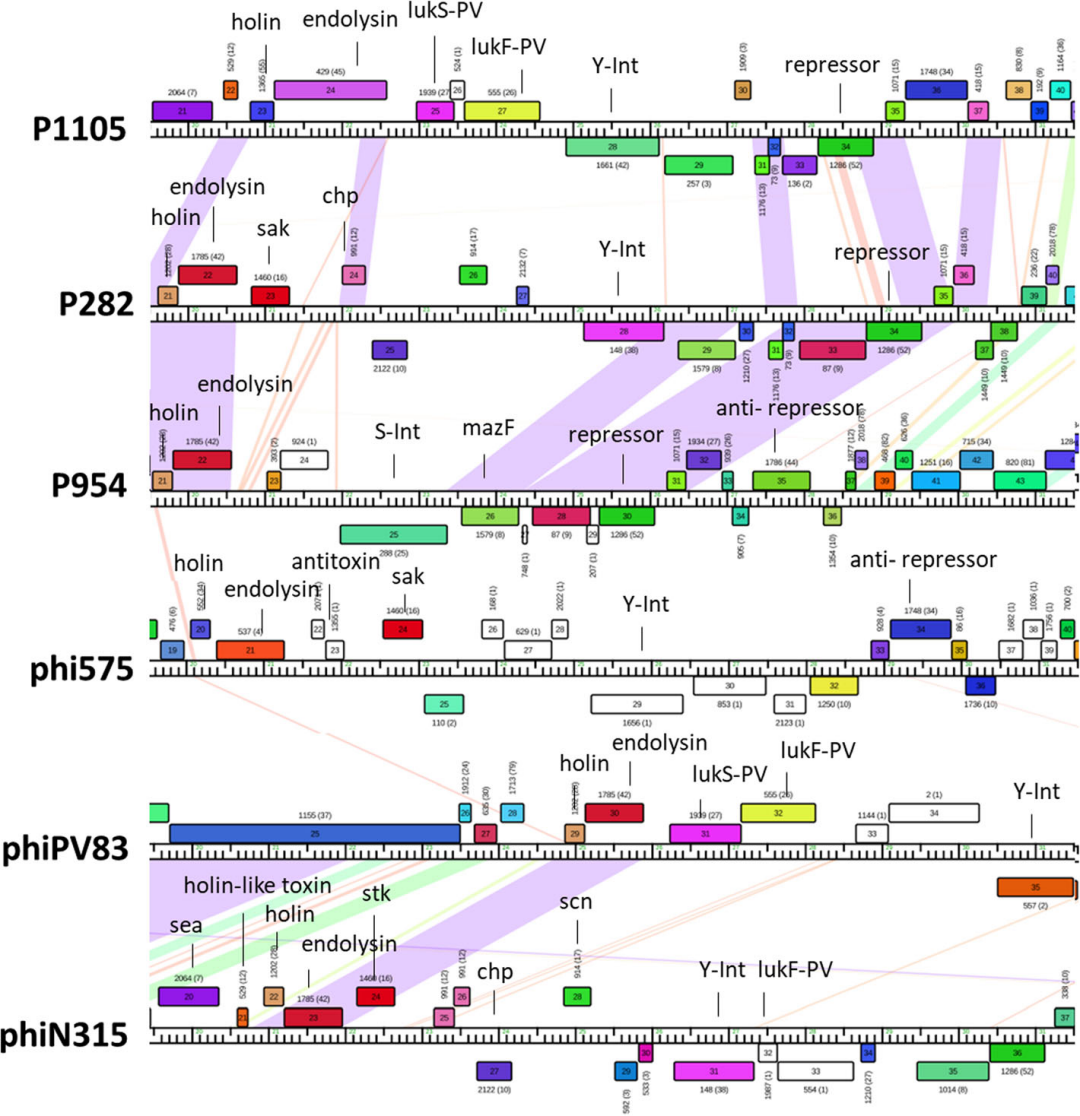
Diversity of staphylococcal phage integrases. Maps of the lysis cassettes, virulence determinants, and integration cassettes for six *Staphylococcus* phages were constructed using Phamerator, genes are labelled with their putative functions where applicable

Temperate phages have the ability to integrate into the bacterial chromosome and reside as prophages. As the unidirectional site-specific integration of phage genome into bacterial chromosome is mediated by integrases, we analysed relationships between the integrase types and Cluster B phages (*n* = 132) that are either temperate or virulent-derivatives of temperate phages; many have been identified as prophages in bacterial genomes (e.g. phi13, phiNM1, phiNM2, phiNM3 and phiNM4) (Fig. 3 and Additional file 34) [21, 28]. We identified integrases in two distinct groups that used either tyrosine or serine as catalytic residues: tyrosine (Y-Int) and serine recombinases (S-Int). Almost all Cluster B staphylococci phages have predicted integrases with the exception of 3A and StB20-like, which likely lost them due to recombination and deletion. The integrases were assigned to five phams; all the serine integrases are members of the same pham, and the tyrosine integrases into the remaining four phams (Fig. 3, Table 1). All of the tyrosine integrases possess a single shared pfam domain (phage_integrase domain, pfam00589), while the S-Int have a different pfam domain in common (C-terminal recombinase, pfam07508). Although Goerke et al. have previously attempted to classify phages according to phage integrases obtaining seven major and eight minor groups [29], our updated dataset demonstrated that no obvious link between type of integrase, host species or subcluster could be made; the same integrase can be detected within phages within different B subclusters and in phages with different hosts. For example, a member of pham 148, which contains the most members within the integrase phams is found in at least one phage from each of the B subclusters, excepting only B1, B11 and B13 (Table 1). The pham with the fewest members, 1656, is found only within a phage in the B8 subcluster, although, other B8 subcluster members contain integrases from a different pham *S. aureus* phage TEM126 contains two predicted integrases, one of each catalytic type, a feature also found in *Gordonia* phages [10]. The roles of the two integrases is unclear. At least five distinct bacterial attachment site (*attB*) sequences, overlapping host tRNA, tmRNA, lipase (*geh*) and β-hemolysin (*hlb*) genes are predicted for phages carrying tyrosine integrase genes (Additional file 34). Collectively, staphylococcal phages exhibit a variety and uncommon number of different site-specific recombinases, like previously observed in *Gordonia*-infecting phages [10].

**Table 1:** Staphylococcal cluster B phage integrases. The dataset includes 205 staphylococcal phages, of which 132 belong to the cluster B *Siphoviridae*. Phams related to integration functions and virulence determinants are represented to phage member, clusters and protein domains

Pham	Function	Alternative nomenclature^a^	Number of members	Domains^b^	Conserved, accessory or unique pham
Integrases					
148	Y-Int	Sa3, Sa9, Sa10, Sa11	38	pfam14659; pfam00589	Conserved (B9); Accessory (B2, B3, B4, B5, B6, B7, B10); Unique (B8, B12, B14, B15, B16, B17)
280	Y-Int	Sa1, Sa5	27	pfam14657; pfam14659; pfam00589	Conserved (B1); Unique (B7); Accessory (B2, B3)
288	S-Int	Sa7, Se1, Se12	25	pfam00239; pfam07508	Accessory (B2, B3, B4); Unique (B6, B10, B11, B13)
1656	Y-Int	–	1	pfam14659; pfam00589	Unique (B8)
1661	Y-Int	Sa2, Sa6	40	pfam00589	Accessory (B3, B5, B6, B7)
Virulence determinants					
297	*virE*		1	pfam05272	Unique (B5)
529	*holin-like*		12	pfam16935	Accessory (B6, B7); Unique (B5)
555	PVL (*lukF-PV*)		26	pfam07968	Accessory (B5, B6, B7)
914	*scn*		17	pfam11546	Accessory (B6, B7); Unique (B3)
1259	*pemK*		10	pfam02452	Accessory (B2, B3); Unique (B5)
1270	*virE*		23	pfam05272	Accessory (B5); Unique (B15)
1322	*holin-like*		1	pfam16935	Unique (B6)
1460	*sak*		16	pfam02821	Accessory (B6, B7); Unique (B8)
1579	*mazF*		8	pfam02452	Accessory (B6)
1597	*hlb*		1	Pfam03372	Unique (B7)
1903	*eta*		5	pfam13365	Accessory (B3); Unique (B2)
1939	PVL (*lukS-PV*)		27	pfam07968	Accessory (B5, B6, B7)
2064	*sea*		7	pfam01123; pfam02876	Accessory (B6)
2122	*chp*		10	pfam11434	Accessory (B6, B7)

^a^ An alternative integrase nomenclature system is provided as in Goerke et al. 2009 (29)

^b^Pham descriptions: pfam14659: Phage integrase, N-terminal SAM-like domain; pfam00589: Phage integrase family; pfam14657: AP2-like DNA-binding integrase domain; pfam00239: Resolvase, N terminal domain; pfam07508: Recombinase; pfam02899: Phage integrase, N-terminal SAM-like domain; pfam13495: Phage integrase, N-terminal SAM-like domain; pfam01123: Staphylococcal/Streptococcal toxin, OB-fold domain; pfam02876: Staphylococcal/Streptococcal toxin, beta-grasp domain; pfam02821: Staphylokinase/Streptokinase family; pfam11434: Chemotaxis-inhibiting protein CHIPS; pfam11546: Staphylococcal complement inhibitor SCIN; pfam05272: Virulence-associated protein E; pfam16935: Putative Holin-like Toxin (Hol-Tox); pfam07968: Leukocidin /Hemolysin toxin family; pfam02452: PemK-like, MazF-like toxin of type II toxin-antitoxin system; pfam13365: Trypsin-like peptidase domain; pfam03372: Endonuclease/Exonuclease/phosphatase family

Acronyms of integrase and virulence genes: Y-Int and S-Int, integrase of tyrosine or serine type; virE, virulence-associated protein E; PVL, Panton-Valentine leucocidin, that is activated by two polypeptide-enconding genes (*lukS-PV*, *lukF-PV*); *scn*, staphylococcal complement inhibitor; *pemK*, endoribonuclease toxin PemK; *sak*, plasminogen activator staphylokinase; *mazF*, endoribonuclease toxin MazF; hlb, β-hemolysin; *eta*, exfoliative toxin A; *sea*, staphylococcal enterotoxin A; *chp*, chemotaxis inhibitory protein

Note: The holin-toxin gene is different from the holin gene that participates in the lytic cassette. For instance, in phage P954, gp20 is the holin-toxin, gp21 is the holing and gp22 is the endolysin

### Virulence genes are exclusively encoded by cluster B phages

*Staphylococcus* prophages have been implicated in the virulence of their hosts through both positive lysogenic conversion, in which prophages encode and express virulence determinants, and through negative lysogenic conversion, in which prophage integration disrupts expression of host encoded virulence associated genes [30]. Prophage interruption of the host β-hemolysin genes (e.g. phi13 and 42E) or lipase (e.g. phiNM4 and IME1346_01) are associated with *S. aureus* virulence [28, 29, 31]. Examples of known prophage-encoded and expressed virulence determinants include the immune-modulator proteins staphylokinase (*sak*), chemotaxis inhibitory protein of *S. aureus* (*chp*) and staphylococcal inhibitor of complement (*scn*) [28], as well as enterotoxin (*sea*), exfoliative (*eta*) and Panton-Valentine leukocidin (*lukF-PV* or *lukS-PV*) toxins (reviewed in [30]).

In our dataset we found that Cluster B phages (*n* = 132) have 14 phams associated with virulence determinant functions (Table 1). Interestingly, the genomic location of almost all virulence determinants is between the cell lysis module and the integrase genes (Fig. 3). The identified phams include the above-mentioned *sak* (pham 1460), *chp* (pham 2122), *scn* (pham 914), *eta* (pham 1903), *sea* (pham 2064) and *lukF-PV* or *lukS-PV* (phams 555 and 1939, respectively), but also the *virE* (pham 297), *holin-like* (pham 529), *pemK* (pham 1259), *mazF* (pham 1579) and *hlb* (pham 1597) toxin genes, whose domains associated with virulence determinant functions are described in Table 1. We note that virulence determinants are highly prevalent and exclusively found in Cluster B phages, although not homogenously distributed throughout the members (Additional file 34). For instance, only ~ 55% of Cluster B phages have identifiable virulence determinants. There are subclusters that don’t have identifiable virulence determinants (B1, B4, B9, B10, B12, B13, B14, B16, and B17), while others have a small (B2 with 32% and B3 with 31%) or a high (B5 with 96%, B6 and B7 with 100%) percentage of phages with virulence determinants and often found in every member (Additional file 34). Phages can either encode one (18%, e.g. SP6), two (14%, e.g. B236), three (14%, e.g. LH1), four (8%, e.g. phiSa119), or even five different virulence determinants (2%, only found in phiN315 and 3 AJ-2017). Based on our Phamerator dataset, only few virulence determinants could be linked to a specific integrase (e.g. phages with Y-int of pham 148 carrying *chp* and and interrupting *hlb*), however, some were subcluster specific; as *virE* (pham 1270) and *sea* (pham 2064) toxins that are associated with subcluster B5 and B6, respectively (Additional file 34). However, we note that genes of unknown function are present in equivalent genome locations in many of the Cluster B phages, and these may represent yet-to-be characterized genes with virulence determinants. Overall, the data obtained reflects the high mosaicism revealed in staphylococci phage genomes and implies a central role of prophages in the evolution and virulence of bacterial pathogens.

### Endolysin genes are organized by different strategies

Phage replication requires a system for progeny release and dispersion to enable new rounds of infection. Multiple strategies, including holin-dependent and holin-independent export to accomplish lysis have been described [32]. The former, typified by phage lambda, is common in dsDNA phages with only some exceptions [33, 34]. In our analysis, we found 12 different holin and 14 different endolysin phams, perhaps having evolved for effective lysis of diverse staphylococcal strains (Additional file 2).

**Fig. 4:**
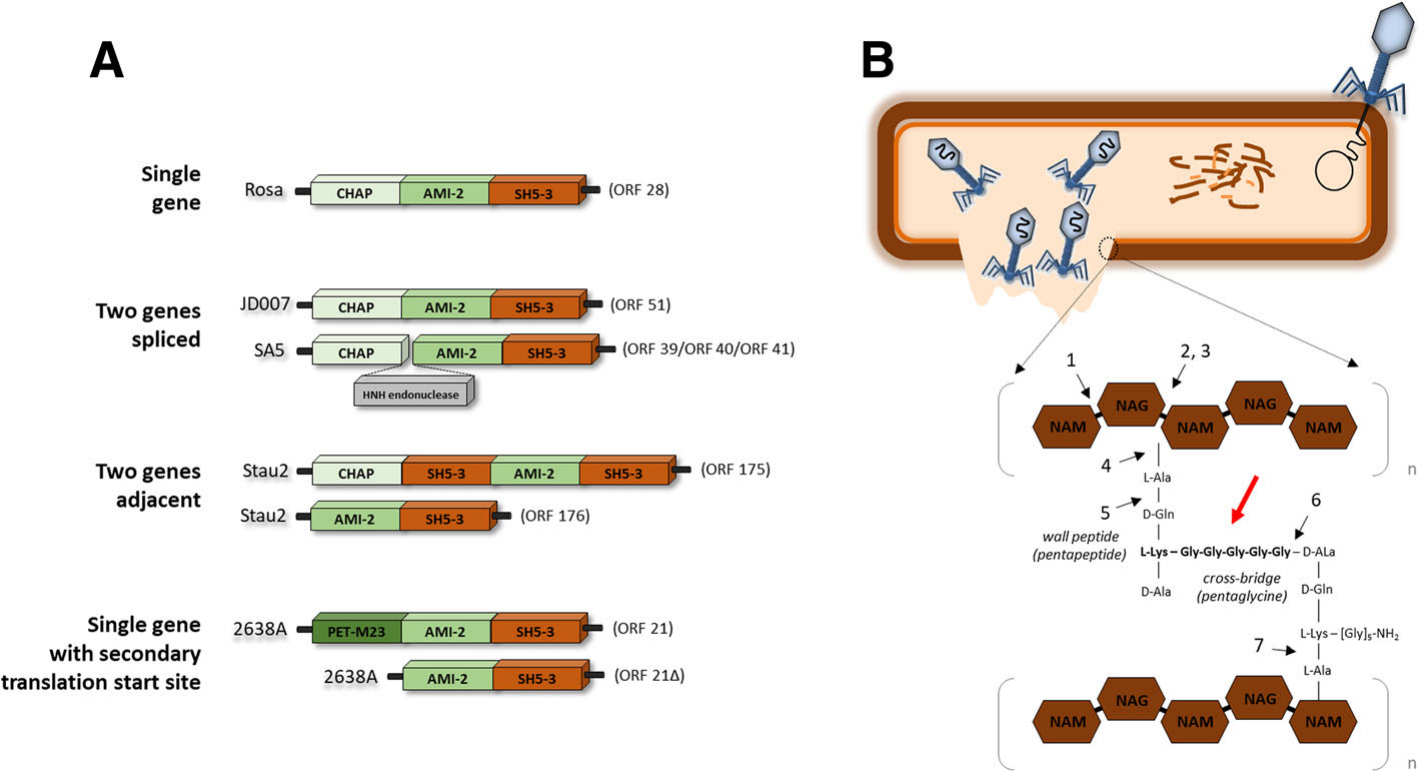
Organization strategies of staphylococcal phage endolysins. **a**) Four endolysin synthesis strategies found in staphylococcal phage genomes (*n* = 205), 175 phages encode lysins with single genes, 20 phage lysin genes exhibit group I introns, nine phages encode lysins using two adjacent genes; one phage, 2638A, uses a single gene with an inter-lytic-domain secondary translational start site, described here [35]; **b**) Schematic representation of a *S. aureus* bacteria cell wall and peptidoglycan. The red arrow indicates the conserved glycine peptidoglycan bridge recognized by the SH3 binding domain. Black arrows indicate possible cutting sites of the catalytic domains: 1) N-acetyl-β-D-glucosaminidase, 2) N-acetyl- β -D-muramidase, 3) lytic transglycosylase, 4) N-acetylmuramoyl- L -alanine amidase, 5) L -alanyl-D-glutamate endopeptidase, 6) D -alanyl-glycyl endopeptidase, 7) L-alanoyl-D-glutamate endopeptidase. Endolysins of class 4) and 6) have been experimental validated [60]. Acronyms: CHAP, cysteine/histidine-dependent amidohydrolase/peptidase domain (PF05257/IPR007921); AMI-2, Amidase_2 domain (PF01510/IPR002502); AMI-3, Amidase_3 domain (PF01520/IPR002508); SH3–5, SH3_5 domain (PF08460/IPR013667); NAG, β-1,4-linked monomers of N-acetyl glucosamine; NAM; N-acetyl muramic acid

We have identified four distinct organization strategies of endolysin genes in the staphylococcal phages (Fig. 4a). From a total of 205 *Staphylococcus* phages, 175 encode endolysins as single genes (e.g. phages 53 and 69), 20 contain group I introns (e.g. phages 85, G1), nine encode endolysins as two adjacent open reading frames (e.g. phages P108, SA11), and one is encoded as a single gene with inter-lytic-domain secondary translation site (phage 2638A) (Additional file 35). The latter endolysin is a unique protein in staphylococcal phage genomes, reported to be expressed as either a endolysin with three lytic domains (a N-terminal peptidase, a centrally located amidase and a C-terminal cell wall binding domain) or a truncated version with only two lytic domains (a N-terminal amidase and a C-terminal cell wall binding domain) [35]. The group I introns have been found in other *Staphylococcus* phages proteins related to morphogenetic and DNA replication, allowing genome recombination and HGT [36]. Of particular interest is also the evolutionary reason behind phages encoding endolysins in two adjacent genes, which suggests that they might act in a cooperative manner to cleave multiple peptidoglycan bonds. Overall, we did not find any particular pattern between the endolysin organization strategy and phage cluster, morphology or host genus.

To understand the molecular and biological basis of these endolysins, we also analysed the presence of functional domains that bind and degrade the staphylococcal peptidoglycan structure (Fig. 4b). Endolysins may contain several catalytic domains responsible for degradation of different bacterial peptidoglycan substrates, the rarest of these being the PET-M23 (peptidase domain M23) and the most frequent the AMI-2 (amidase 2 domain), AMI-3 (amidase 3 domain) and CHAP (cysteine- and histidine-dependent amidohydrolase/peptidase, present in 96% of the endolysins) (Additional file 35). Conversely, there is only one binding domain belonging to the SH3 family of proteins identified in ∼96% of the endolysins, that recognises the peptidoglycan substrate, demonstrating a conserved recognition strategy of endolysins to the staphylococcal peptidoglycan polymer.

## Discussion

We describe here the comparative genomic analysis of 205 staphylococci phages isolated at different times and from different geographical regions. *S. aureus* phages have been previously classified based on morphology and serology [37], or comparative genomics [38, 39]. In 2012, shared gene content based on BLASTP alignments and reciprocal shared matches was used to compute a distance matrix of mostly *S. aureus*-infecting phages (*n* = 85), revealing three distinct groups matching different morphologies: class I (podoviruses), class II (siphoviruses) and class III (myoviruses), class II was further divided into nine subclusters [19]. In this study, we updated and revised this classification with an additional dataset of genomes (*n* = 205) and including phages infecting other species of *Staphylococcus* genus. Major contributions to our updated dataset came from phages infecting coagulase-negative staphylococci, which were limited to three phages in the previous analysis (Additional file 1). Following the same division, we have also found three clusters (A, B and C that correspond to class I, II and III, respectively) but resolved one additional Cluster D (6ec and vB_SepS_SEP9) delineating a group of distinct siphoviruses as well as the singleton (SPbeta-like) (Fig. 1). We also provide a higher resolution of the genetic diversity by subdividing clusters A and B into several new subclusters that emerged by reorganizing of old and recently deposited phages (Additional file 1, Additional files 6, 7, 8, 9, 10, 11, 12, 13, 14, 15, 16, 17, 18, 19, 20, 21, 22, 23, 24, 25, 26, 27, 28, 29, 30, 31, 32, 33). The low proportion of singletons found in our phage dataset (n = 205, 0.5% are singletons), contrasts with higher numbers found in phages infecting hosts of similar taxonomic level, such as *Bacillus* (*n* = 83, 18.1% are singletons) [14] and *Gordonia* phages (*n* = 79, 17.7% are singletons) [10], indicating that the analysed staphylococci phages represent fewer phage types, possibly due to restrictive sampling conditions and methods used. Nonetheless, we show that there is high genetic diversity of staphylococci phage genomes leading to the numerous cluster/subclusters resolved and ORF phamilies obtained (*n* = 2139, where the largest pham has only 101 members) (Additional file 2). Most important would be the isolation of the undersampled coagulase-negative-infecting viruses to reveal the presence of new genes and relationships that shape the evolution of staphylococci phage genomes [40].

Like the Actinobacteriophages, [13, 41, 42], *Staphylococcus* phage genomes are mosaic in nature, due to horizontal exchange, deletion and addition of ORFs and ORF modules between members (e.g. Additional file 13), likely driven by non-homologous recombination including transposases [43], endonucleases [44] and site-specific recombinases [45] (Additional file 2). Analysis of shared gene content for this dataset via the MaxGCDGap metric (~ 0.33, Fig. 2d) that show low levels of discontinuity across of the spectrum of possible phage genetic relationships, also hints at the active participation of phages in HGT. Moreover, the high number and diversity of phams (n = 2139, of which 745 are orphams and without conserved domains) (Additional file 2), also suggest a large influx of genes across novel bacterial hosts and/or other phages by HGT, similar to what was observed in *Pseudomonas* phages [42].

The genetic relationships of the phages described here reflect a general model for phage evolution in which host-virus dynamics have driven diversity over a long period of evolutionary time. Although we have described many distinct lineages with low levels of nucleotide sequence similarity, they have not evolved completely independently, and there are many incidents of horizontal genetic exchange between lineages. These are observed as homologues – albeit distantly related – residing in distinct genomic context in different genomes. Such HGT events are likely to occur when the phages – or their ancestors – are present in the same host species, but the constant pressure to avoid resistance by switching to different hosts confers an impediment to HGT and the emergence of distinct lineages.

Similar to Actinobacteria phages [10], staphylococci Cluster B phages display a large array of lysogenic modules, including multiple integrases, and likely use a variety of *attP* sites (Table 1 and Additional file 34); a temperate lifestyle has been experimentally demonstrated for several members (e.g. phiNM3, phiNM4, vB_SepiS-phiIPLA5 and vB_SepiS-phiIPLA7) [21, 46, 47]. Many of these phages are associated with the presence of virulence determinants, which provides the bacterial hosts with additional genes and competitive advantages. For instance, the PVL-carrying phage phiSLT (toxin that causes leukocytolysis and tissues necroris) that lysogenize several PVL-negative *S. aureus* strains using the same 29-bp *attB*, that subsequently acquire the ability to express the PVL toxin, is an example of prophage contribution for the dissemination and evolution of pathogenicity in staphylococci [47]. The common location of virulence determinants downstream of lysis module (Fig. 3) suggests that expression is coupled to phage induction, a scenario that was experimentally validated for phi13 and other PLV toxin-carrying phages [48, 49]; however, some virulence determinants may also be expressed from the prophage, as observed for Cluster N temperate mycobacteriophages [50]. In contrast, some phages have the ability to silence several host proteins associated with virulence determinant functions via disruption of these genes through integration during lysogenization, as it is demonstrated by phage phi13 and the 5′-end of the β-hemolysin gene (51). However, this loss is usually accompanied with acquisition of new virulence determinants in the staphylococcal chromosome. Thus, by characterizing the current set of staphylococci phage genomes available at the GenBank, we demonstrate a high diversity of integrases and reported novel virulence-associated genes (e.g. *hbl* and *virE*) [29], showing a potential for more a versatile role of phages in the adaptive evolution of staphylococcal bacterial pathogens. However, because we have limited our dataset to the complete phage genomes deposited at GenBank at the time of writing, there is a still the potential for additional diverse prophage-derived integrases/virulence determinant genes to be found in staphylococcal genomes. A future similar analysis may identify additional virulence determinants, particularly in phages related to those underrepresented in our current study.

Staphylococci phage endolysin genes are organized in four different manners (single gene, two genes spliced, two genes adjacent and single gene with inter-lytic-domain secondary translational start site), which results in a predicted unusual complex expression system observed in phage genomes (Fig. 4 and Additional file 35). The endolysin functional domains include several catalytic domains but share a single SH3 binding domain (Additional file 35). It has been proposed that staphylococcal phage endolysin SH3 binds to the peptidoglycan pentaglycine cross-bridge [51]. Lysostaphin (a bacteriocin from *Staphylococcus simulans* bv. *staphylolyticus*) and its homologue, hydrolase ALE-1, also display a SH3 domain that recognizes the same epitope (69, 70). Therefore, we speculate that SH3 domains of *Staphylococcus* phage endolysins similarly target glycine-rich bridges identified in 11 out of 12 staphylococci species represented in our study. Despite the conservation of the binding domain, not all of these endolysins are likely to degrade a broad spectrum of staphylococci when added exogenously to liquid cultures, as is observed for endolysins of phages SAP-2 and K, [52, 53], as the endolysin of vB_SauM-LM12 endolysin is specific to *S. aureus* [54]. Additional characterization of the endolysins is required to determine the specificity of the catalytic domains with respect to host strain [54]. Overall, the maintenance of the SH3 domain despite the various endolysin modular organizations, is a likely response to environmental pressures.

## Conclusions

In summary, by offering a high-resolution and updated view of the staphylococcal viral genetic diversity as well as gene flux patterns within and across different phage groups (cluster and subclusters) we provide novel insights into their evolution. Future biotechnological applications include development of integration-dependent vectors for construction of recombinant staphylococcal strains; and genetic engineering of endolysins for both detection and control of staphylococcal bacterial pathogens.

## Methods

### Virus metadata collection

Biopython 32 package was used within the conda environment (https://www.continuum.io) to retrieve fully sequenced *Staphylococcus* phage genomes deposited at GenBank as of June 2018 (*n* = 205) and to create the FASTA files. Python functions scripts were also used to collect and list in a table the important features, such as host taxonomy, genome size, GC content, number of ORFs, number of tRNAs, viral taxonomy for each RefSeq record. All metadata retrieved is given in the Additional file 1. Python code created is accessible upon request. During our analysis we excluded the following phages: PT1028 (NC_007045.1), SA1 (NC_027991.1), SpaA1 (NC_018277.1), HOB 14.1.R1 (CP018841.1), pSco-10 (KX011028.1), IME1367_02 (KY653121.1), IME1364_01 (KY653128.1) and SA7 (KY695153.1), for being incomplete or wrongly deposited as *Staphylococcus*-infecting viruses. Due to strong genomic evidence, we corrected phage UPMK_2 morphology from *Podoviridae* to *Siphoviridae*.

### Average nucleotide identity values metric

Average Nucleotide Identity (ANI) values from whole-genome pairwise comparisons were generated with Kalign [55]. Heat map was created in Excel. This was used as the first of a total three metrics used to characterized phage genomes.

### Shared gene content metric

All staphylococcal phage genomes were analysed with Phamerator to group genes into phams - gene products of related proteins [56]. The number of phams generated was used as a second metric to determine phage grouping. First, a SQL database was created locally and customized for incorporation of information present in GenBank files. Second, phage genomes were imported into Phamerator to assign phams with kclust. Phamerator identifies conserved domains in all genes using the NCBI conserved domain database [57]. Analysis were performed on Intel-based PCs with the Windows 7 operating system with a Virtual Machine (Oracle VM Virtual Box) running the Ubuntu 16.04 operating system for execution of Phamerator python scripts in Linux command line. Data manipulation and adjustments in the database scheme were made with MySQL language queries. All the python code created is accessible upon request.

SplitsTree network was used to visualize the relationship of shared gene content between staphylococcal phage genomes [58, 59]. Phams generated by Phamerator were scored by the presence/absence. Protein repertoire relatedness was used in SplitsTree to visualize and analysed the evolutionary data, using network functionality.

### Gene content dissimilarity metric

The gene content dissimilarities (GCDs) metric was used to further explored phage relationships [10, 11]. GCD was computed for each pairwise comparison to calculate the number of shared phams between the two divided by the total number of phams present in each genome. The two proportions were also averaged and converted to a gene content dissimilarity: where GCD = 1 means 100% dissimilar (no shared phams) and GCD = 0 complete similar (all shared phams). Plots were generated using GCD versus number of pairwise comparisons. The GCD formula is:

$$ \mathrm{GCD}=1\hbox{-} \left(\frac{\frac{\mathrm{Shared}\ \mathrm{phams}}{\mathrm{Total}\ \mathrm{phams}\ \mathrm{in}\ \mathrm{genome}\ \mathrm{A}}+\frac{\mathrm{Shared}\ \mathrm{phams}}{\mathrm{Total}\ \mathrm{phams}\ \mathrm{in}\ \mathrm{genome}\ \mathrm{B}}}{2}\right) $$

Phage-specific MaxGCDGap distances were calculated as previously described [10]. For each phage, all pairwise GCD values were ranked by magnitude, and the difference between each consecutive GCD value was calculated (GCD gap). GCD gap is defined the following formula: GCDgap _(n, n + 1)_ = GCD_n_-GCD _n + 1_. Gap ranges from near 0 (indicating small gene content discontinuities) to 1 (indicating large gene content discontinuities). The MaxGCDGap is the largest of these values. Plots were generated using MaxGCDGap versus pairwise comparisons ordered by magnitude. All GCD related data was calculated with custom written python scripts [11].

### Cluster assignment

Cluster assignment was based on shared gene content. A cut-off of 35% of shared genes (phams) was used to place phages solely in one cluster, a metric recently used to assigned *Gordonia* phages [10].

## Additional files

Additional file 1: Staphylococcal phage genome characteristics. Biopython package was used to retrieve complete genome sequences at June of 2018 from NCBI and list in a table with several features associated to the phage. *Numbers in parentheses indicate the size of DNA regions (in bp) homologous to regions of known terminal repeats. DTR, direct terminal repeat; COS, cohesive end site. CoPS, coagulase-positive species, CoVS, coagulase-variable species, CoNS, coagulase-negative species. (XLSX 29 kb)

Additional file 2: Phams. The dataset includes 205 staphylococcal phages, encoding 20,579 predicted ORFs, a total of 2139 phams (gene with related sequences) of which 745 orphams (genes without related sequences) were identified generated based on kclust alignments performed in Phamerator. Phams are ordered by highest conservation among phage members. The phage-ORF column reflects the positioning of genes locus of the genome map generated by Phamerator. Biopython functions were used to retrieved and list in a table with several features associated to the phages. * An alternative nomenclature system for Cluster B phage integrases is provided as in Goerke et al. 2009 (29). (XLSX 216 kb)

Additional file 3: Average nucleotide sequence identity values. Average nucleotide sequence identities (ANIs) of 205 staphylococcal phage genomes were made using Kalign algorithm. Heat map was created in excel. (XLSX 41 kb)

Additional file 4: Shared gene content. Biopython functions were used to assignment gene content similarity using the Phamerator output (2139 phams, of which 745 are orphams). Heat map was created in excel. (XLSX 223 kb)

Additional file 5: Conserved, accessory and unique phams assigned to each subcluster. The distribution of a) Cluster A, b) Cluster B, c) Cluster C and d) Cluster D proteins. Conserved phams are conserved among all members (back). Accessory phams are shared by at least two members (grey). Unique phams are singletons (white). Subclusters B7, B9, B11-B12, B14-B17, C4 and D2 and singleton (SPbeta-like) represented by one member are not shown. Subclusters A2, B13, C5-C6 and D1 represented by two members have no accessory proteins. While there conserved phams among the subclusters can be directly visualized here, phams shared by different groups can be consulted in Additional file 2. As shown in Venn Diagram provided in Fig. 1, there is no universal pham in the staphylococci phage genomes. (PDF 567 kb)

Additional file 6: Whole-genome map of subcluster A1 phages. Maps were generated using Phamerator in which pairwise sequence similarity (minimal BLASTN cut-off E value is 10^− 4^) is given according to colour spectrum (purple and red lines denote regions of highest and lowest nucleotide similarity, respectively). Ruler corresponds to genome base pairs. Proteins are labelled with predicted function and given a specific colour (shared phams i.e. gene members have the same colour, orphams i.e. unique genes are shown in white). Gene numbering reflects the re-organization of genomes give here to start with packaging genes or at defined ends (all gene related information can be consulted in Additional file 2), and their positioning above or below the bar correspond to rightwards or leftwards transcription, respectively. (PDF 61 kb)

Additional file 7: Whole-genome map of subcluster A2 phages. Represented as mentioned above. (PDF 20 kb)

Additional file 8: Whole-genome maps of subcluster B1 phages. Represented as mentioned above. (PDF 87 kb)

Additional file 9: Whole-genome map of subcluster B2 phages. Represented as mentioned above. (PDF 205 kb)

Additional file 10: Whole-genome map of subcluster B3 phages. Represented as mentioned above. (PDF 284 kb)

Additional file 11: Whole-genome map of subcluster B4 phages. Represented as mentioned above. (PDF 111 kb)

Additional file 12: Whole-genome map of subcluster B5 phages. Represented as mentioned above. (PDF 271 kb)

Additional file 13: Whole-genome maps of subcluster B6 phages. Represented as mentioned above. (PDF 195 kb)

Additional file 14: Whole-genome map of subcluster B7 phages. Represented as mentioned above. (PDF 136 kb)

Additional file 15: Whole-genome map of subcluster B8 phages. Represented as mentioned above. (PDF 37 kb)

Additional file 16: Whole-genome map of subcluster B9 phages. Represented as mentioned above. (PDF 35 kb)

Additional file 17: Whole-genome map of subcluster B10 phages. Represented as mentioned above. (PDF 50 kb)

Additional file 18: Whole-genome map of subcluster B11 phages. Represented as mentioned above. (PDF 23 kb)

Additional file 19: Whole-genome maps of subcluster B12 phages. Represented as mentioned above. (PDF 24 kb)

Additional file 20: Whole-genome map of subcluster B13 phages. Represented as mentioned above. (PDF 36 kb)

Additional file 21: Whole-genome map of subcluster B14 phages. Represented as mentioned above. (PDF 24 kb)

Additional file 22: Whole-genome map of subcluster B15 phages. Represented as mentioned above. (PDF 15 kb)

Additional file 23: Whole-genome map of subcluster B16 phages. Represented as mentioned above. (PDF 23 kb)

Additional file 24: Whole-genome maps of subcluster B17 phages. Represented as mentioned above. (PDF 24 kb)

Additional file 25: Whole-genome map of subcluster C1 phages. Represented as mentioned above. (PDF 1182 kb)

Additional file 26: Whole-genome map of subcluster C2 phages. Represented as mentioned above. (PDF 182 kb)

Additional file 27: Whole-genome map of subcluster C3 phages. Represented as mentioned above. (PDF 170 kb)

Additional file 28: Whole-genome map of subcluster C4 phages. Represented as mentioned above. (PDF 37 kb)

Additional file 29: Whole-genome map of subcluster C5 phages. Represented as mentioned above. (PDF 72 kb)

Additional file 30: Whole-genome map of subcluster C6 phages. Represented as mentioned above. (PDF 76 kb)

Additional file 31: Whole-genome map of subcluster D1 phages. Represented as mentioned above. (PDF 55 kb)

Additional file 32: Whole-genome map of subcluster D2 phages. Represented as mentioned above. (PDF 35 kb)

Additional file 33: Whole-genome map of subcluster singleton SPbeta-like. Represented as mentioned above. (PDF 40 kb)

Additional file 34: Integrases, *attB* sites and virulence genes. The dataset includes all cluster B staphylococcal siphoviruses (*n* = 132), for which five integrases types, five *attB* sites and 13 virulence factors were identified. The integrases were identified through Phamerator and BLASTP. The *attB* site was found through BLASTP using best host species hit similar to the one originally used to isolate the phage. The virulence genes were retrieved from Additional file 2 and re-organized. (XLSX 72 kb)

Additional file 35: Endolysin genes. The dataset includes 205 staphylococcal phages, for which four distinct organization strategies were found (single gene, two genes spliced, two genes adjacent and single gene with inter-lytic-domain secondary translational start site). The corresponding nucleotide and amino acid sequences and functional encoding domains (catalytic and binding) detected through the structural database HHpred are given. All genes spliced with group I introns were manually curated. (XLSX 99 kb)

## Abbreviations

ANI: Average nucleotide identity
attB: Bacterial attachment site
chp: Chemotaxis inhibitory protein
GCD: Gene content dissimilarity
geh: Lipase
HGT: Horizontal gene transfer
hlb: β-hemolysin
Int: Integrase of tyrosine (Y-Int) or serine (S-Int) type
lukS-PV and lukF-PV: Panton-Valentine bi-component leukocidin subunits S or F
mazF: Endoribonuclease toxin MazF
sak: Plasminogen activator staphylokinase
sea: Staphylococcal enterotoxin A
snc: Staphylococcal complement inhibitor
