# Supplementary figures and images for: Staphylococci phages display vast genomic diversity and evolutionary relationships
Source: BMC Genomics. 2019 May 9;20:357. doi: 10.1186/s12864-019-5647-8 (PMC6507118; doi:10.1186/s12864-019-5647-8)

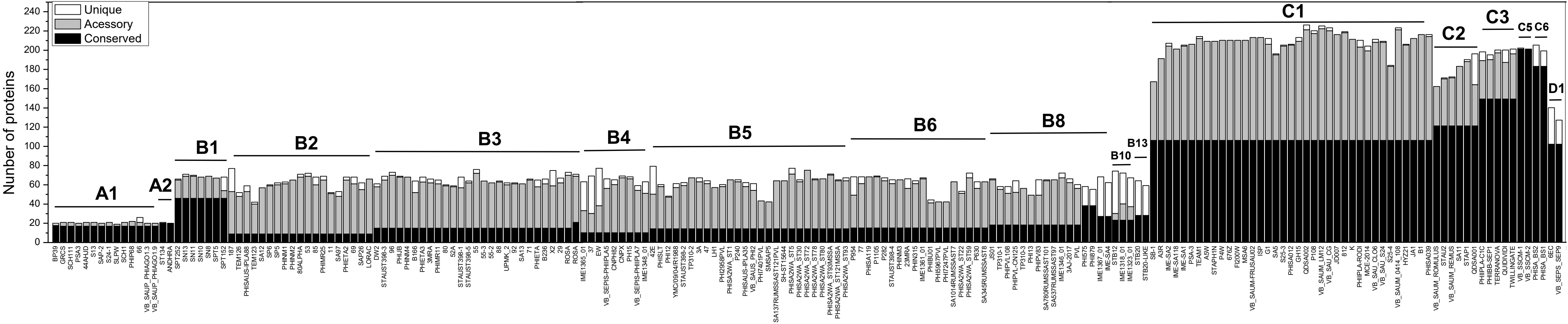

Conserved phams

Cluster A

A1 - [12, 185, 245, 353, 432, 513, 584, 699, 712, 887, 1528, 1729, 1785, 1801, 1900, 2009, 2103]  
A2 - [12, 54, 245, 307, 336, 353, 432, 513, 537, 580, 584, 1528, 1659, 1689, 1729, 1801, 1807, 1900, 1998, 2103]

Cluster B

B1 - [6, 13, 22, 77, 82, 91, 105, 126, 158, 237, 280, 489, 670, 671, 674, 691, 709, 744, 776, 820, 825, 839, 868, 869, 879, 994, 1007, 1022, 1119, 1156, 1180, 1202, 1297, 1366, 1384, 1385, 1410, 1455, 1495, 1659, 1662, 1701, 1785, 1933, 2012, 2059]  
B2 - [105, 744, 1022, 1156, 1309, 1455, 1701, 1933, 2012]  
B3 - [414, 468, 546, 556, 579, 655, 832, 1015, 1151, 1156, 1680, 1695, 1701, 1713, 1852]  
B4 - [21, 50, 414, 434, 546, 556, 1680, 1701, 2012, 2107]  
B5 - [179, 829, 857, 879, 915, 1204, 1234, 1328, 1562, 1626, 1658, 1746, 1852, 1969]  
B6 - [179, 371, 538, 635, 732, 743, 842, 858, 1019, 1142, 1155, 1286, 1657, 1725, 1805]  
B7 - [17, 337, 446, 468, 576, 635, 731, 743, 842, 849, 874, 1147, 1194, 1248, 1454, 1497, 1635, 1932]  
B8 - [17, 39, 51, 110, 222, 330, 446, 489, 537, 552, 676, 700, 715, 716, 743, 842, 938, 985, 1007, 1017, 1042, 1147, 1155, 1160, 1194, 1220, 1250, 1256, 1380, 1454, 1497, 1517, 1518, 1635, 1748, 1912, 1932, 1941]  
B9 - [148, 236, 386, 446, 507, 559, 743, 787, 820, 829, 843, 849, 962, 1077, 1160, 1170, 1194, 1372, 1377, 1407, 1443, 1485, 1496, 1765, 1766, 1977, 2138]  
B10 - [ 104, 105, 237, 308, 670, 744, 820, 846, 1022, 1156, 1326, 1336, 1455, 1531, 1583, 1638, 1701, 1740, 1914, 1933, 1968, 2011, 2012]  
B13 - [201, 211, 251, 259, 333, 339, 446, 552, 645, 654, 724, 731, 849, 884, 1077, 1194, 1204, 1414, 1496, 1695, 1746, 1796, 1806, 1935, 1942, 1977, 2011, 2121]

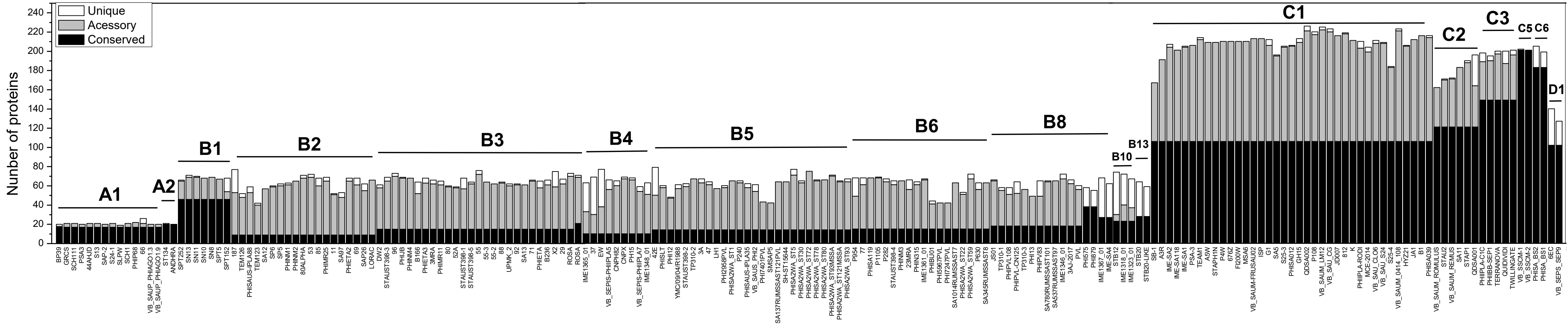

## Conserved phams

### Cluster C

C1 - [14, 44, 62, 76, 84, 107, 108, 115, 121, 127, 150, 165, 210, 246, 262, 277, 279, 289, 291, 325, 373, 397, 404, 417, 445, 458, 464, 472, 496, 511, 570, 581, 583, 621, 633, 639, 697, 713, 723, 733, 758, 766, 777, 809, 812, 859, 865, 879, 884, 922, 961, 986, 992, 1079, 1080, 1088, 1093, 1101, 1114, 1143, 1150, 1192, 1254, 1257, 1262, 1276, 1279, 1281, 1346, 1395, 1413, 1448, 1451, 1465, 1467, 1477, 1522, 1582, 1593, 1598, 1606, 1625, 1634, 1644, 1650, 1669, 1726, 1727, 1737, 1836, 1845, 1861, 1923, 1954, 1962, 1973, 1975, 1980, 2010, 2026, 2027, 2032, 2051, 2069, 2074, 2090]

C2 - [14, 20, 31, 32, 44, 62, 84, 108, 121, 146, 150, 187, 188, 210, 253, 262, 279, 289, 298, 325, 397, 401, 464, 472, 477, 511, 540, 551, 561, 570, 583, 639, 687, 713, 733, 741, 758, 779, 809, 812, 824, 838, 859, 879, 888, 922, 963, 982, 992, 1079, 1080, 1088, 1101, 1107, 1112, 1143, 1148, 1149, 1150, 1162, 1165, 1192, 1199, 1254, 1257, 1262, 1276, 1279, 1281, 1291, 1346, 1370, 1413, 1422, 1448, 1451, 1465, 1466, 1467, 1477, 1513, 1522, 1541, 1542, 1593, 1599, 1632, 1634, 1644, 1645, 1650, 1669, 1688, 1727, 1739, 1741, 1782, 1795, 1834, 1835, 1842, 1857, 1901, 1923, 1927, 1960, 1962, 1973, 1974, 2010, 2026, 2027, 2032, 2063, 2069, 2074, 2087, 2105, 2115, 2129, 2134]

C3 - [14, 32, 44, 63, 84, 106, 107, 108, 121, 127, 146, 150, 164, 188, 210, 246, 262, 277, 279, 289, 298, 325, 349, 350, 397, 417, 445, 464, 467, 472, 477, 511, 541, 566, 570, 581, 583, 586, 633, 639, 654, 661, 682, 703, 713, 751, 758, 763, 809, 812, 823, 836, 838, 859, 884, 910, 922, 936, 961, 970, 979, 982, 986, 992, 1010, 1079, 1088, 1106, 1107, 1108, 1131, 1143, 1149, 1150, 1182, 1189, 1193, 1199, 1207, 1213, 1254, 1262, 1279, 1281, 1305, 1307, 1346, 1364, 1367, 1368, 1413, 1430, 1448, 1451, 1462, 1465, 1466, 1467, 1473, 1474, 1477, 1520, 1522, 1537, 1542, 1567, 1593, 1599, 1609, 1625, 1632, 1634, 1637, 1640, 1644, 1669, 1710, 1727, 1732, 1739, 1774, 1814, 1832, 1836, 1857, 1891, 1901, 1923, 1962, 1964, 1973, 1975, 1990, 1993, 2010, 2026, 2027, 2028, 2030, 2032, 2038, 2051, 2056, 2069, 2086, 2089, 2105, 2116, 2131]

C5 - [3, 14, 16, 18, 20, 25, 32, 35, 44, 62, 75, 84, 107, 108, 121, 125, 134, 142, 146, 147, 150, 165, 178, 181, 183, 188, 210, 225, 232, 246, 262, 279, 282, 304, 316, 325, 340, 360, 361, 376, 382, 397, 402, 403, 409, 439, 445, 447, 455, 456, 457, 464, 472, 477, 481, 496, 508, 511, 517, 548, 549, 562, 570, 581, 583, 586, 604, 621, 633, 660, 669, 697, 703, 713, 723, 727, 733, 757, 758, 784, 795, 803, 809, 812, 837, 838, 851, 859, 877, 879, 899, 906, 907, 917, 918, 922, 927, 931, 937, 992, 1053, 1066, 1067, 1079, 1088, 1093, 1101, 1113, 1120, 1122, 1141, 1150, 1177, 1179, 1199, 1241, 1252, 1254, 1257, 1269, 1276, 1281, 1294, 1301, 1302, 1313, 1314, 1331, 1346, 1364, 1395, 1413, 1421, 1435, 1448, 1451, 1465, 1466, 1467, 1477, 1478, 1488, 1493, 1510, 1521, 1522, 1534, 1538, 1539, 1542, 1560, 1593, 1595, 1599, 1601, 1605, 1620, 1621, 1625, 1628, 1634, 1637, 1642, 1644, 1650, 1652, 1669, 1687, 1691, 1710, 1727, 1739, 1752, 1771, 1782, 1791, 1836, 1884, 1901, 1923, 1959, 1962, 1971, 1973, 1975, 1994, 2010, 2024, 2025, 2026, 2027, 2028, 2032, 2041, 2069, 2084, 2105, 2106, 2108, 2116, 2117]

C6 - [3, 8, 14, 34, 44, 48, 63, 89, 99, 108, 115, 121, 125, 145, 146, 150, 156, 194, 210, 216, 225, 242, 261, 262, 269, 275, 277, 279, 289, 298, 309, 323, 341, 373, 376, 397, 399, 403, 417, 445, 447, 453, 456, 464, 472, 473, 477, 492, 496, 502, 508, 510, 511, 515, 528, 540, 548, 570, 583, 611, 628, 678, 682, 713, 723, 726, 733, 757, 758, 809, 812, 823, 826, 838, 852, 859, 878, 879, 888, 894, 902, 922, 925, 937, 942, 961, 968, 979, 992, 998, 1000, 1028, 1034, 1049, 1079, 1088, 1093, 1094, 1132, 1133, 1150, 1165, 1199, 1207, 1225, 1252, 1254, 1276, 1278, 1285, 1293, 1298, 1334, 1344, 1348, 1349, 1351, 1382, 1412, 1421, 1424, 1431, 1448, 1451, 1465, 1466, 1467, 1477, 1478, 1483, 1487, 1542, 1591, 1599, 1624, 1625, 1630, 1634, 1637, 1644, 1649, 1663, 1674, 1678, 1685, 1726, 1727, 1739, 1741, 1769, 1782, 1783, 1837, 1857, 1868, 1888, 1899, 1901, 1923, 1957, 1962, 1973, 1975, 1981, 1982, 1985, 1989, 2010, 2027, 2028, 2029, 2032, 2034, 2069, 2070, 2081, 2096, 2097, 2105, 2115, 2116, 2124, 2135]

### Cluster D

D1 - [1, 26, 93, 106, 113, 127, 151, 159, 164, 166, 169, 176, 202, 219, 310, 315, 374, 385, 433, 448, 449, 480, 521, 545, 552, 560, 646, 651, 657, 688, 692, 706, 725, 730, 742, 789, 799, 814, 840, 861, 882, 890, 959, 986, 1026, 1076, 1120, 1131, 1168, 1169, 1172, 1183, 1200, 1201, 1203, 1204, 1209, 1242, 1272, 1329, 1333, 1368, 1403, 1413, 1415, 1463, 1473, 1481, 1494, 1501, 1509, 1547, 1581, 1633, 1651, 1694, 1699, 1711, 1746, 1754, 1768, 1792, 1803, 1814, 1823, 1844, 1878, 1936, 1949, 1952, 1955, 1967, 2005, 2006, 2036, 2045, 2054, 2055, 2067, 2068, 2085, 2121]

Supplement: Supplementary file 5 — Conserved, accessory and unique phams assigned to each subcluster. The distribution of a) Cluster A, b) Cluster B, c) Cluster C and d) Cluster D proteins. Conserved phams are conserved among all members (back). Accessory phams are shared by at least two members (grey). Unique phams are singletons (white). Subclusters B7, B9, B11-B12, B14-B17, C4 and D2 and singleton (SPbeta-like) represented by one member are not shown. Subclusters A2, B13, C5-C6 and D1 represented by two members have no accessory proteins. While there conserved phams among the subclusters can be directly visualized here, phams shared by different groups can be consulted in Additional file 2. As shown in Venn Diagram provided in Fig. 1, there is no universal pham in the staphylococci phage genomes. (PDF 567 kb) [file 12864_2019_5647_MOESM5_ESM.pdf]

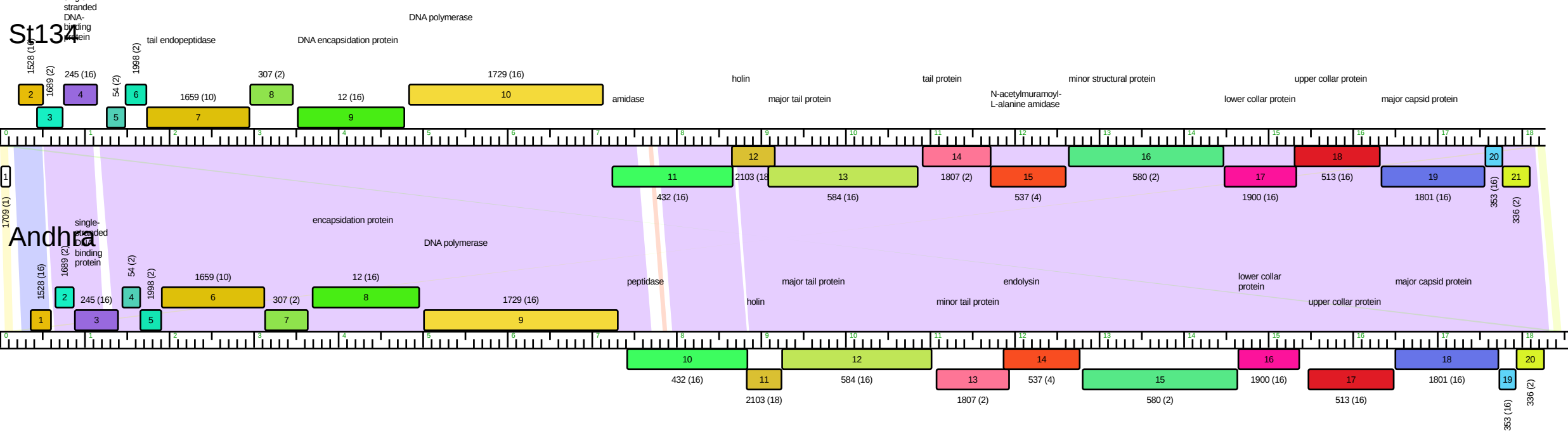

Supplement: Supplementary file 7 — Whole-genome map of subcluster A2 phages. Represented as mentioned above. (PDF 20 kb) [file 12864_2019_5647_MOESM7_ESM.pdf]

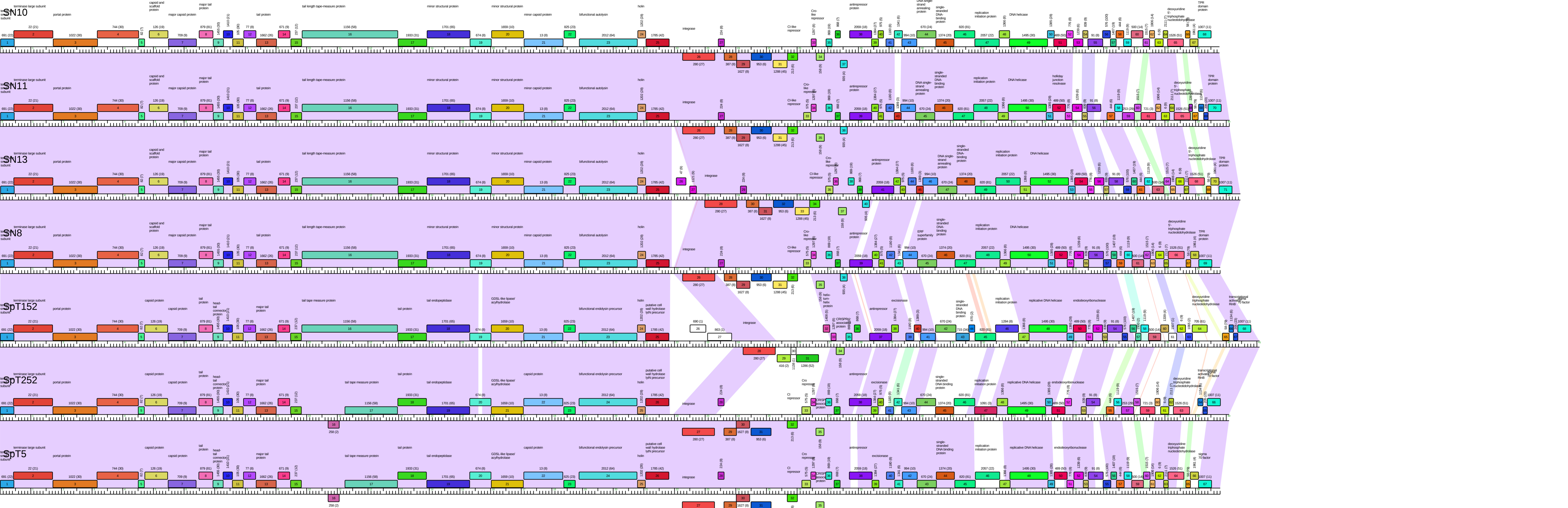

Supplement: Supplementary file 8 — Whole-genome maps of subcluster B1 phages. Represented as mentioned above. (PDF 87 kb) [file 12864_2019_5647_MOESM8_ESM.pdf]

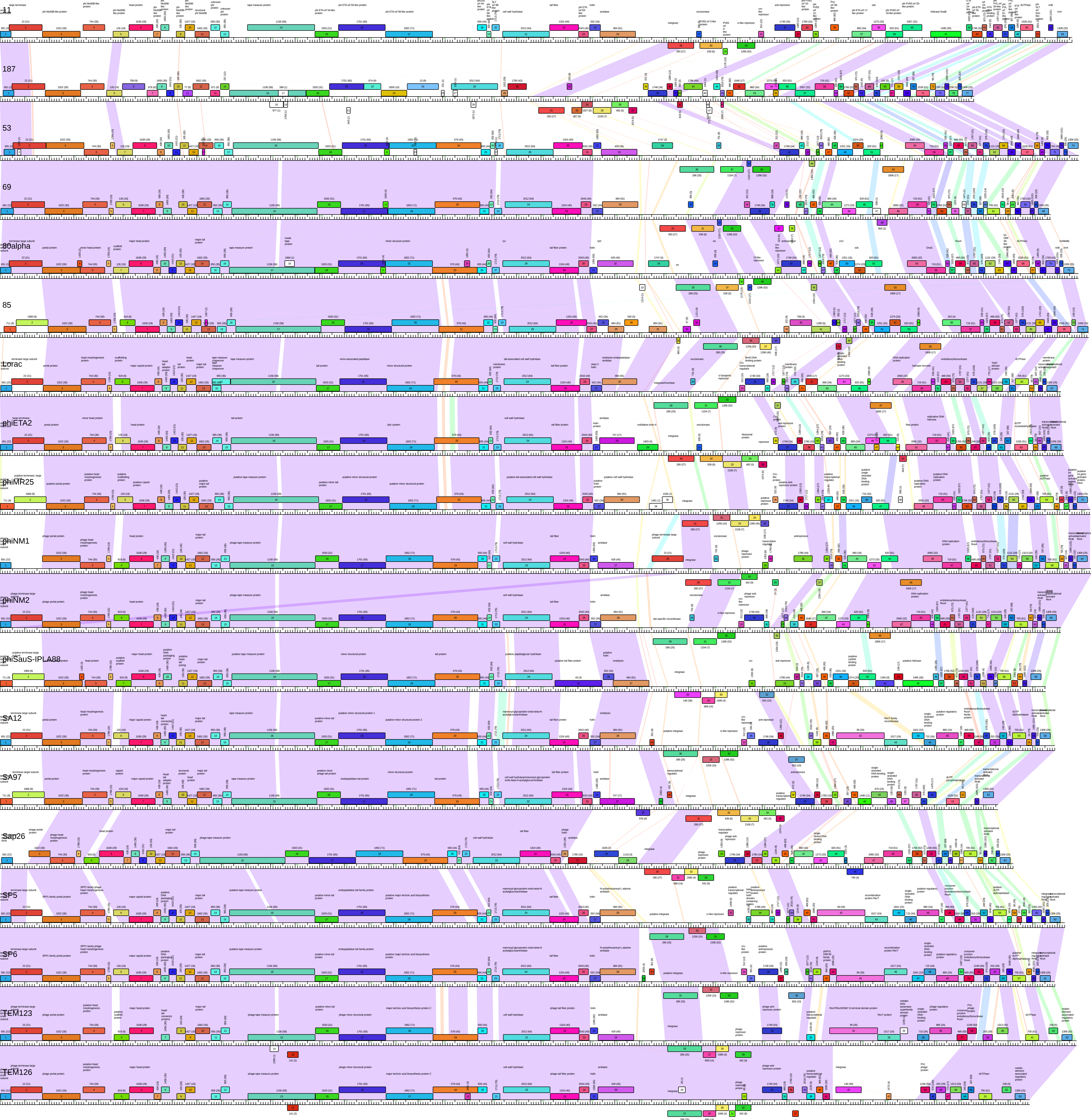

Supplement: Supplementary file 9 — Whole-genome map of subcluster B2 phages. Represented as mentioned above. (PDF 205 kb) [file 12864_2019_5647_MOESM9_ESM.pdf]

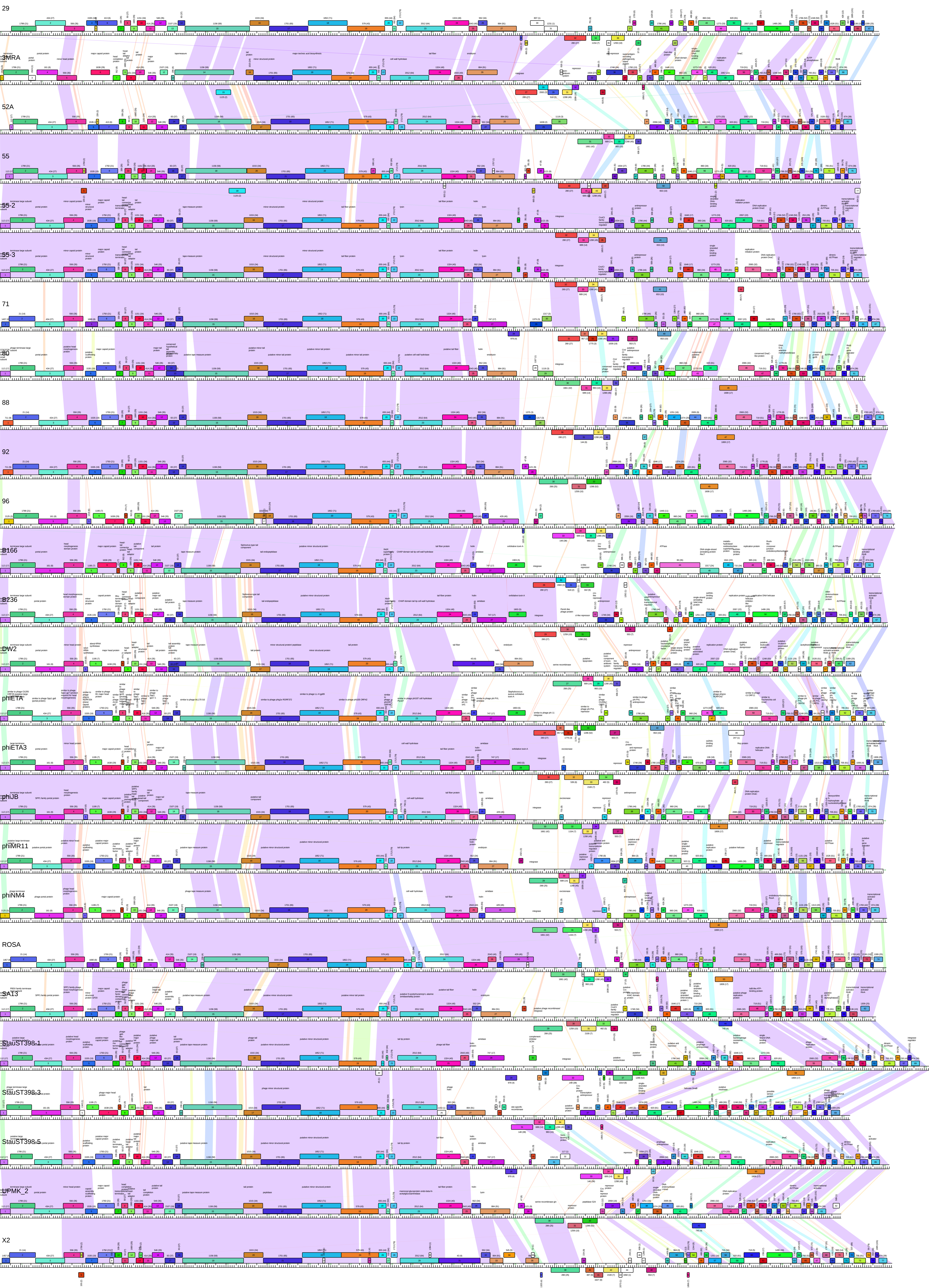

Supplement: Supplementary file 10 — Whole-genome map of subcluster B3 phages. Represented as mentioned above. (PDF 284 kb) [file 12864_2019_5647_MOESM10_ESM.pdf]

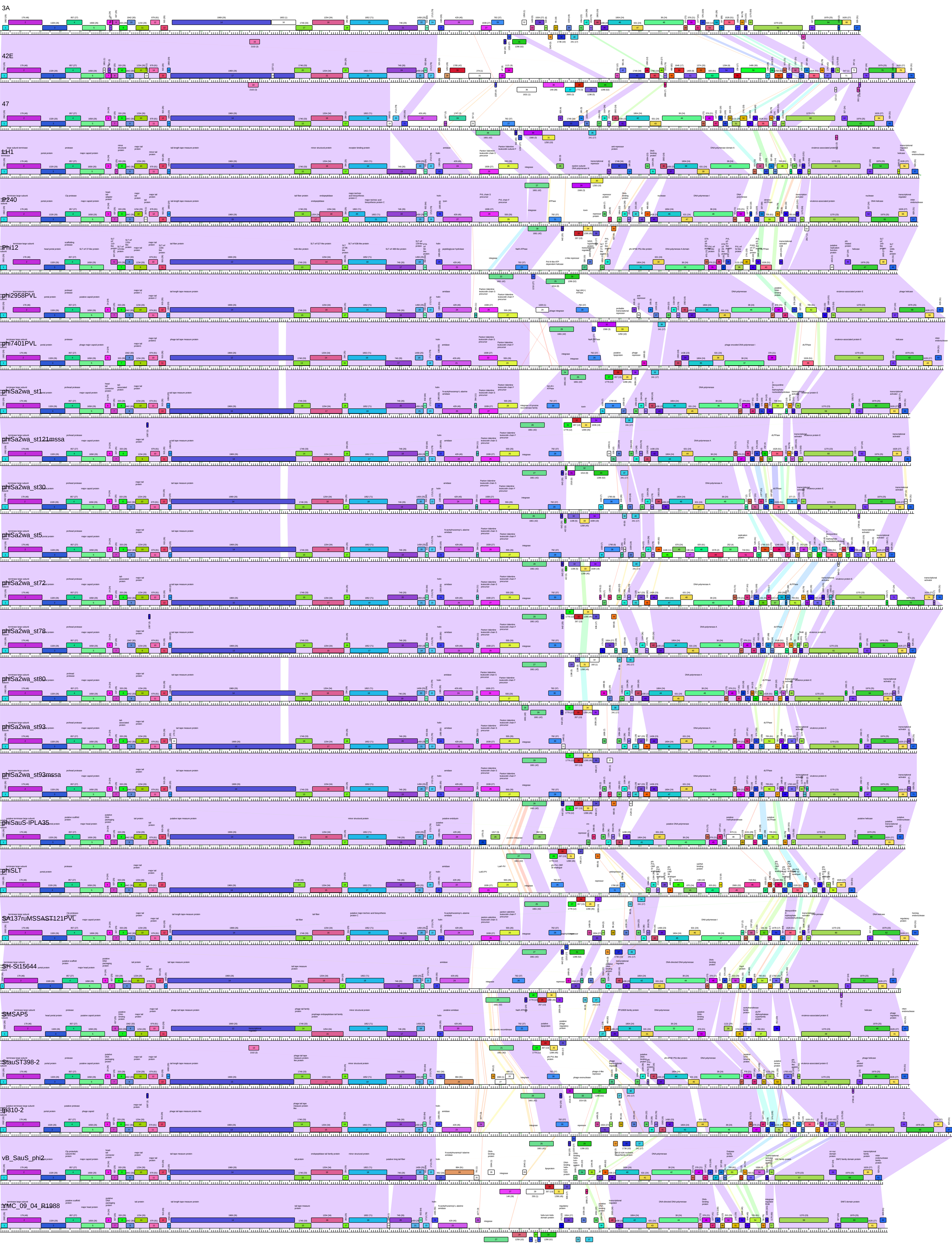

Supplement: Supplementary file 12 — Whole-genome map of subcluster B5 phages. Represented as mentioned above. (PDF 271 kb) [file 12864_2019_5647_MOESM12_ESM.pdf]

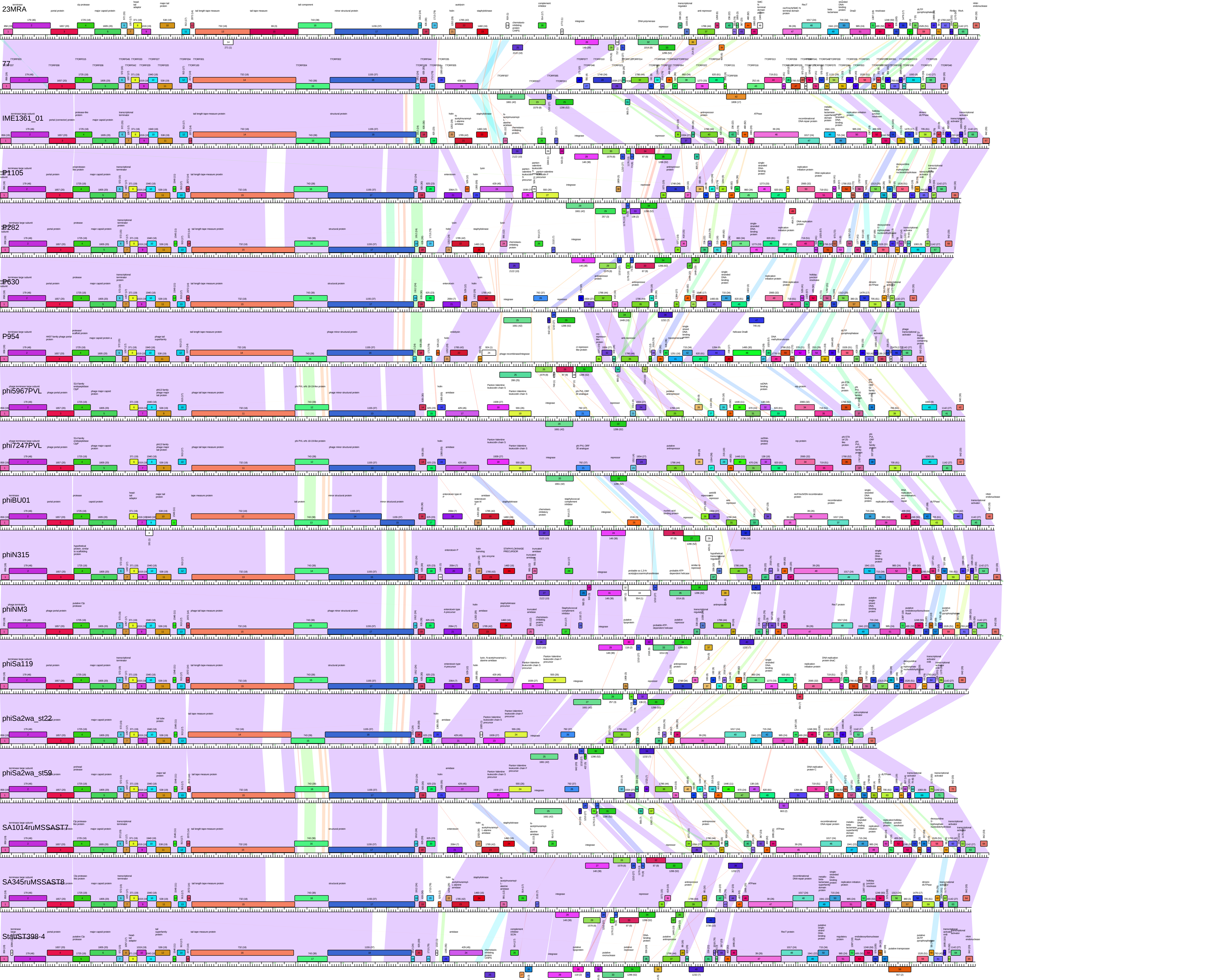

Supplement: Supplementary file 13 — Whole-genome maps of subcluster B6 phages. Represented as mentioned above. (PDF 195 kb) [file 12864_2019_5647_MOESM13_ESM.pdf]

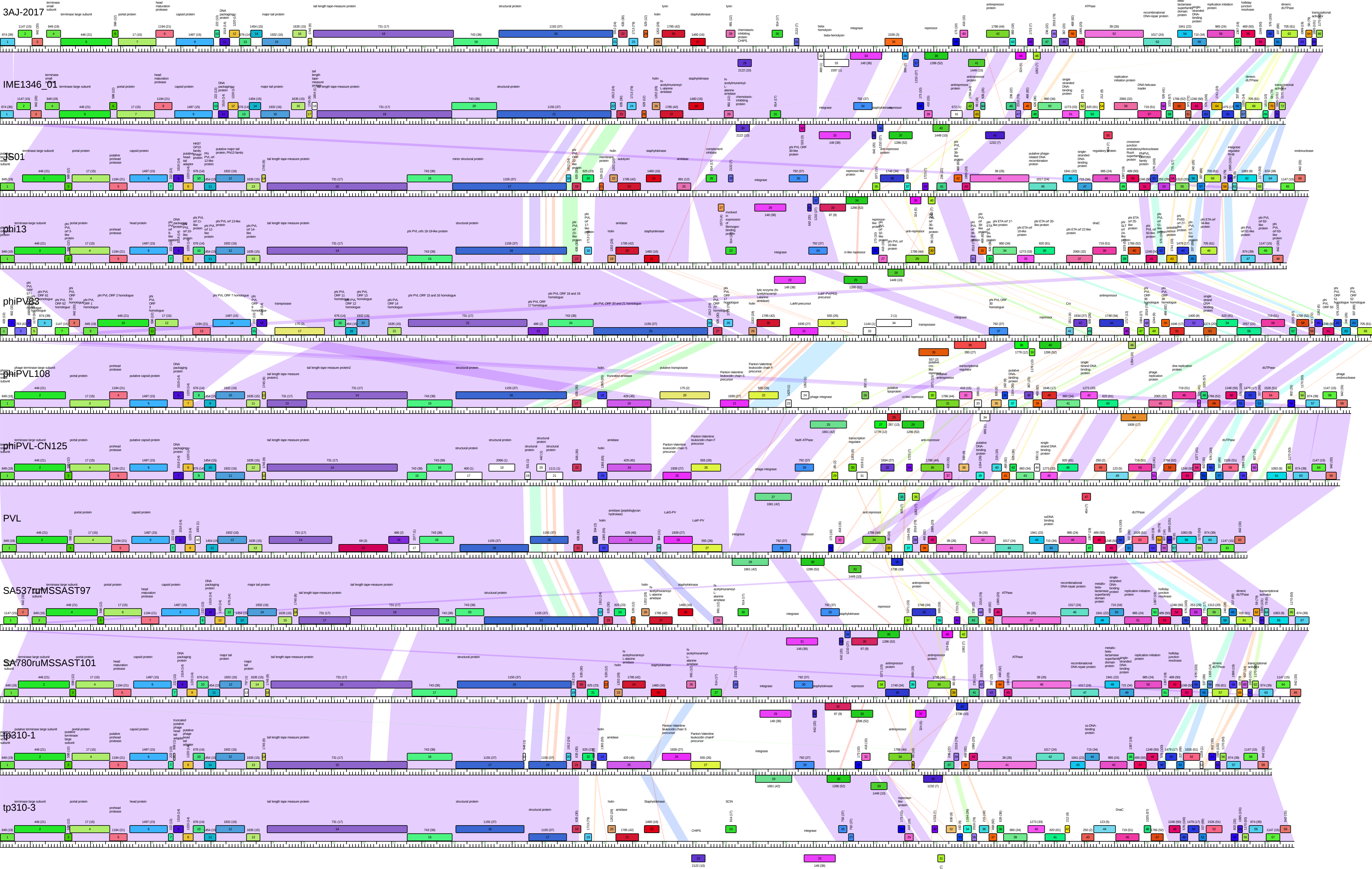

Supplement: Supplementary file 14 — Whole-genome map of subcluster B7 phages. Represented as mentioned above. (PDF 136 kb) [file 12864_2019_5647_MOESM14_ESM.pdf]

ph1575

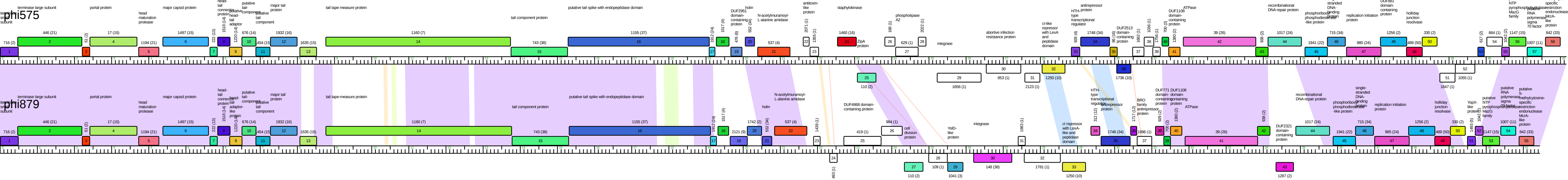

ph1879

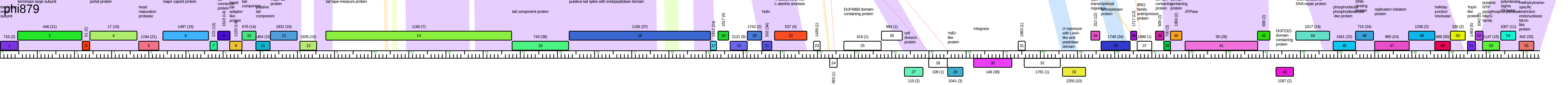

Supplement: Supplementary file 15 — Whole-genome map of subcluster B8 phages. Represented as mentioned above. (PDF 37 kb) [file 12864_2019_5647_MOESM15_ESM.pdf]

ME1367\_01

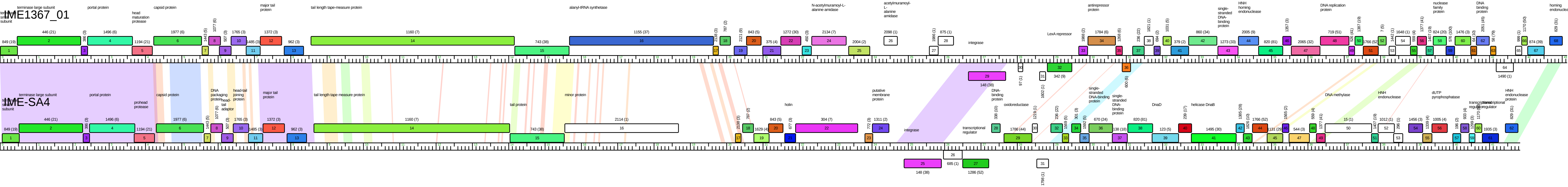

ME-SA4

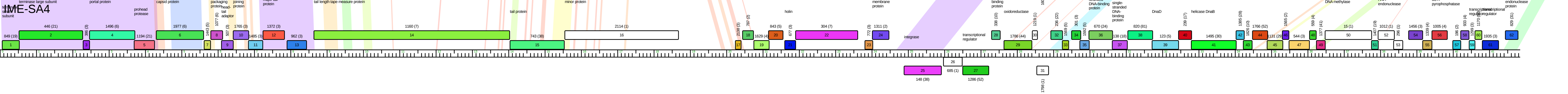

Supplement: Supplementary file 16 — Whole-genome map of subcluster B9 phages. Represented as mentioned above. (PDF 35 kb) [file 12864_2019_5647_MOESM16_ESM.pdf]

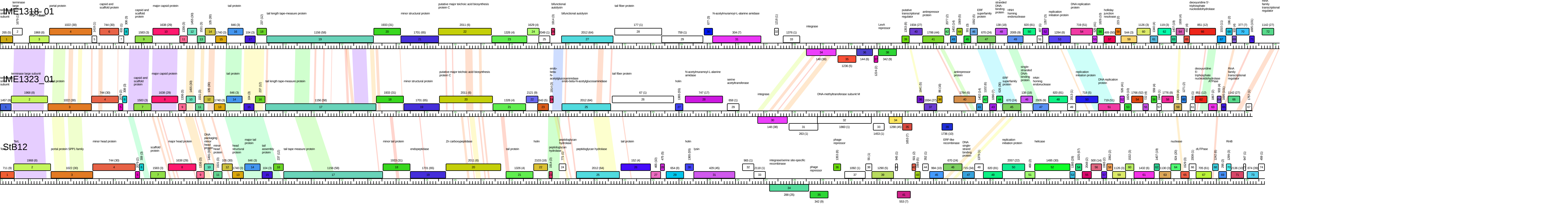

Supplement: Supplementary file 17 — Whole-genome map of subcluster B10 phages. Represented as mentioned above. (PDF 50 kb) [file 12864_2019_5647_MOESM17_ESM.pdf]

TerL  
StB27  
TerS

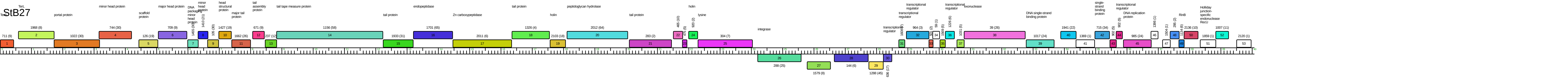

Supplement: Supplementary file 18 — Whole-genome map of subcluster B11 phages. Represented as mentioned above. (PDF 23 kb) [file 12864_2019_5647_MOESM18_ESM.pdf]

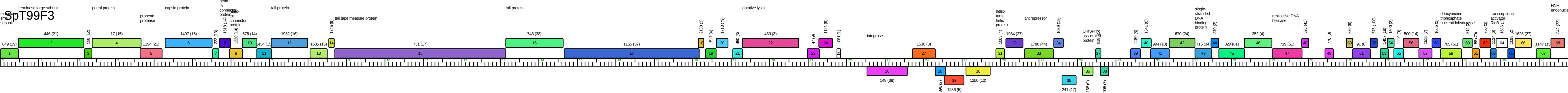

Supplement: Supplementary file 19 — Whole-genome maps of subcluster B12 phages. Represented as mentioned above. (PDF 24 kb) [file 12864_2019_5647_MOESM19_ESM.pdf]

# phIRs7

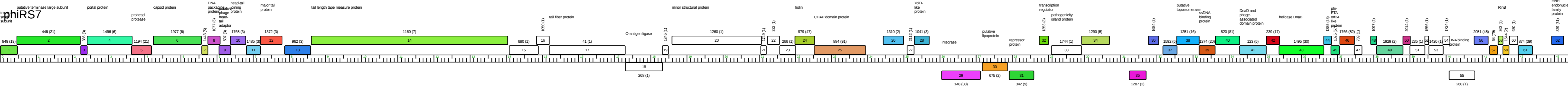

Supplement: Supplementary file 21 — Whole-genome map of subcluster B14 phages. Represented as mentioned above. (PDF 24 kb) [file 12864_2019_5647_MOESM21_ESM.pdf]

2638A

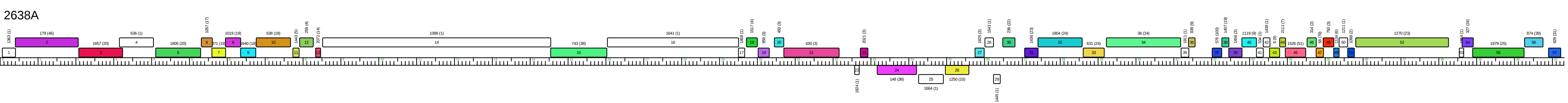

Supplement: Supplementary file 22 — Whole-genome map of subcluster B15 phages. Represented as mentioned above. (PDF 15 kb) [file 12864_2019_5647_MOESM22_ESM.pdf]

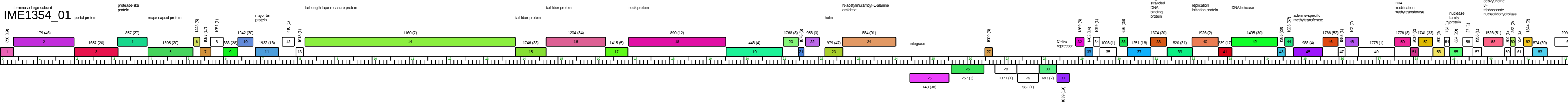

Supplement: Supplementary file 23 — Whole-genome map of subcluster B16 phages. Represented as mentioned above. (PDF 23 kb) [file 12864_2019_5647_MOESM23_ESM.pdf]

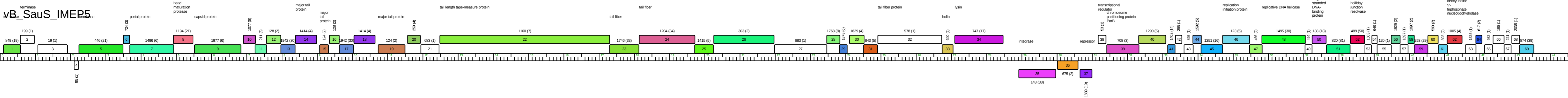

Supplement: Supplementary file 24 — Whole-genome maps of subcluster B17 phages. Represented as mentioned above. (PDF 24 kb) [file 12864_2019_5647_MOESM24_ESM.pdf]

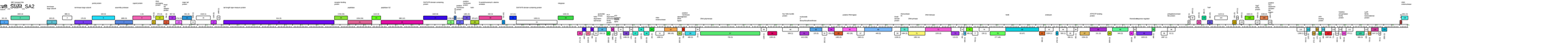

Supplement: Supplementary file 32 — Whole-genome map of subcluster D2 phages. Represented as mentioned above. (PDF 35 kb) [file 12864_2019_5647_MOESM32_ESM.pdf]
