# Supplementary material for: Staphylococci phages display vast genomic diversity and evolutionary relationships
Source: BMC Genomics. 2019 May 9;20:357. doi: 10.1186/s12864-019-5647-8 (PMC6507118; doi:10.1186/s12864-019-5647-8)
Supplement: Supplementary file 6 — Whole-genome map of subcluster A1 phages. Maps were generated using Phamerator in which pairwise sequence similarity (minimal BLASTN cut-off E value is 10− 4) is given according to colour spectrum (purple and red lines denote regions of highest and lowest nucleotide similarity, respectively). Ruler corresponds to genome base pairs. Proteins are labelled with predicted function and given a specific colour (shared phams i.e. gene members have the same colour, orphams i.e. unique genes are shown in white). Gene numbering reflects the re-organization of genomes give here to start with packaging genes or at defined ends (all gene related information can be consulted in Additional file 2), and their positioning above or below the bar correspond to rightwards or leftwards transcription, respectively. (PDF 61 kb) [file 12864_2019_5647_MOESM6_ESM.pdf]

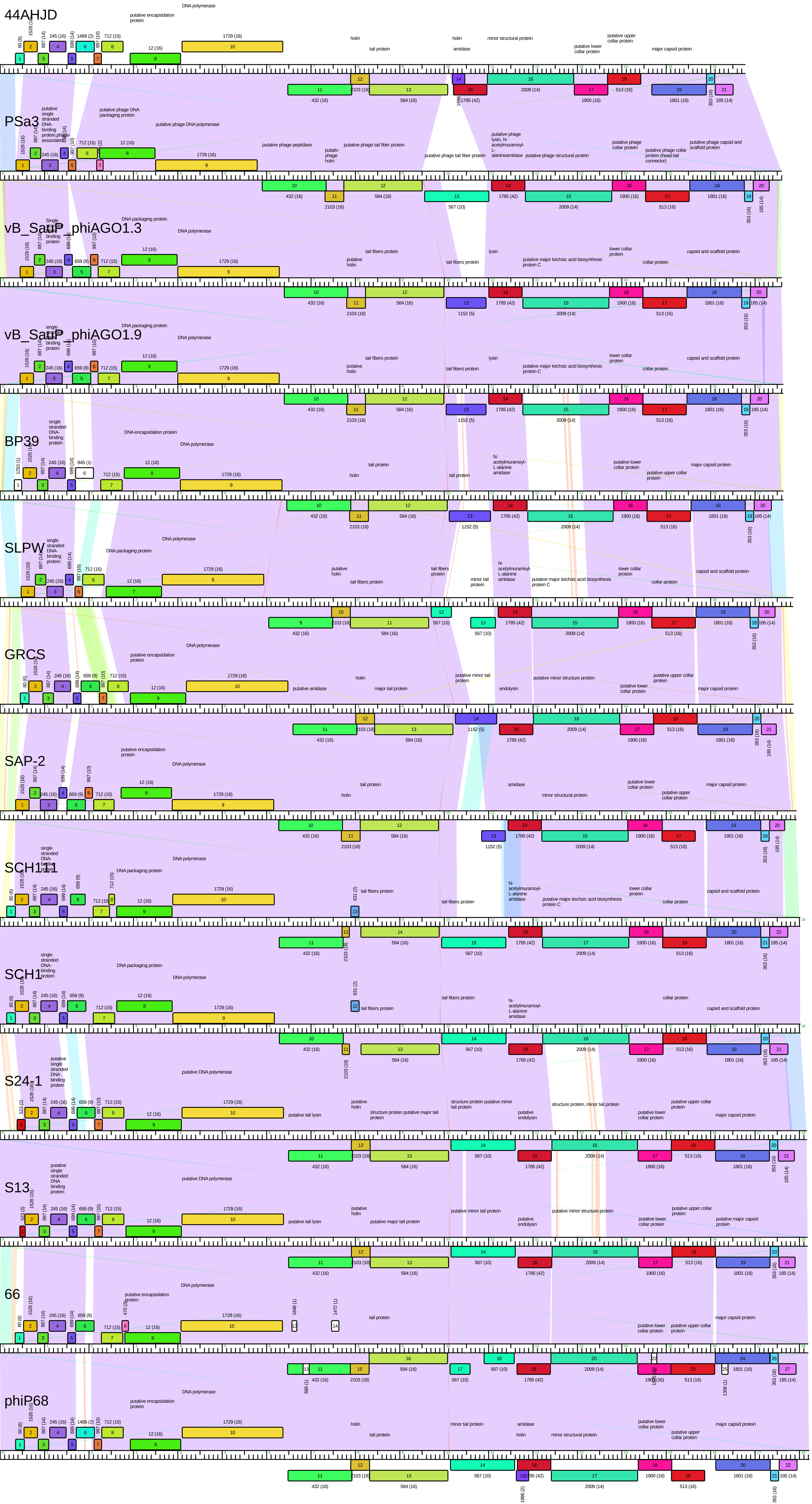

44AHJD
putative encapsidation protein
DNA polymerase
holin
tail protein
amidase
minor structural protein
putative lower collar protein
putative upper collar protein
major capsid protein
PSa3
putative single stranded DNA-binding protein,phage associated
putative phage DNA packaging protein
putative phage DNA polymerase
putative phage peptidase
putative phage tail fiber protein
putative phage lysin, N-acetylmuramoyl-L-alanineamidase
putative phage structural protein
putative phage collar protein
putative phage collar protein (head-tail connector)
putative phage capsid and scaffold protein
vB_SauP_phiAGO1.3
DNA packaging protein
DNA polymerase
tail fibers protein
putative holin
lysin
putative major teichoic acid biosynthesis protein C
lower collar protein
collar protein
capsid and scaffold protein
vB_SauP_phiAGO1.9
BP39
single stranded DNA-binding protein
DNA encapsidation protein
N-acetylmuramoyl-L-alanine amidase
putative upper collar protein
SLPW
minor tail protein
GRCS
putative amidase
major tail protein
putative minor tail protein
endolysin
putative minor structure protein
SAP-2
SCH111
SCH1
S24-1
putative DNA polymerase
putative tail lysin
structure protein putative major tail protein
structure protein putative minor tail protein
putative endolysin
structure protein, minor tail protein
putative major capsid protein
S13
putative major tail protein
putative minor tail protein
putative minor structure protein
66
phiP68
