# Supplementary material for: Staphylococci phages display vast genomic diversity and evolutionary relationships
Source: BMC Genomics. 2019 May 9;20:357. doi: 10.1186/s12864-019-5647-8 (PMC6507118; doi:10.1186/s12864-019-5647-8)
Supplement: Supplementary file 11 — Whole-genome map of subcluster B4 phages. Represented as mentioned above. (PDF 111 kb) [file 12864_2019_5647_MOESM11_ESM.pdf]

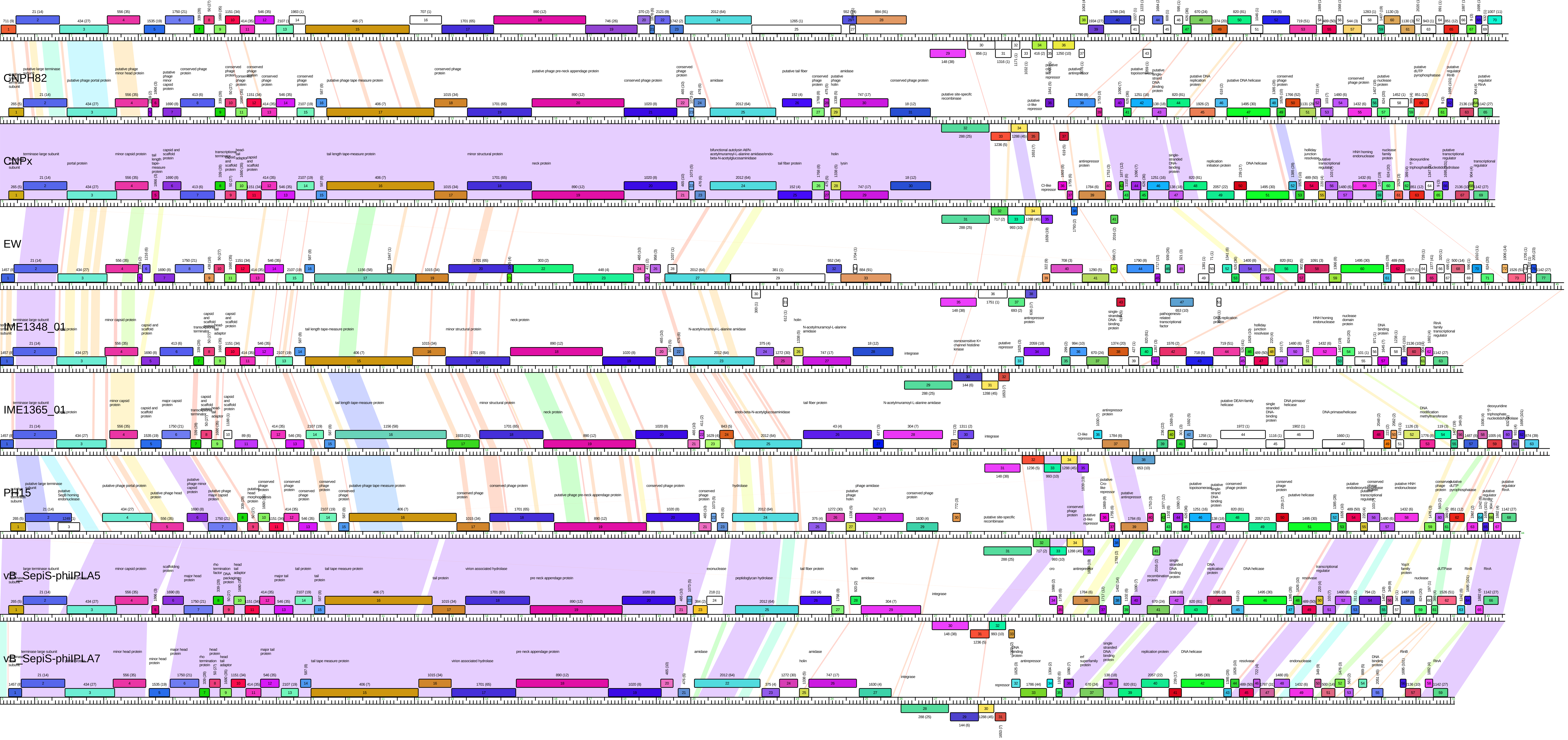
CNPH82
CNPx
EW
IME1348_01
IME1365_01
PH15
vB_SepiS-phiIPLA5
vB_SepiS-phiIPLA7
