# Supplementary material for: Staphylococci phages display vast genomic diversity and evolutionary relationships
Source: BMC Genomics. 2019 May 9;20:357. doi: 10.1186/s12864-019-5647-8 (PMC6507118; doi:10.1186/s12864-019-5647-8)
Supplement: Supplementary file 20 — Whole-genome map of subcluster B13 phages. Represented as mentioned above. (PDF 36 kb) [file 12864_2019_5647_MOESM20_ESM.pdf]

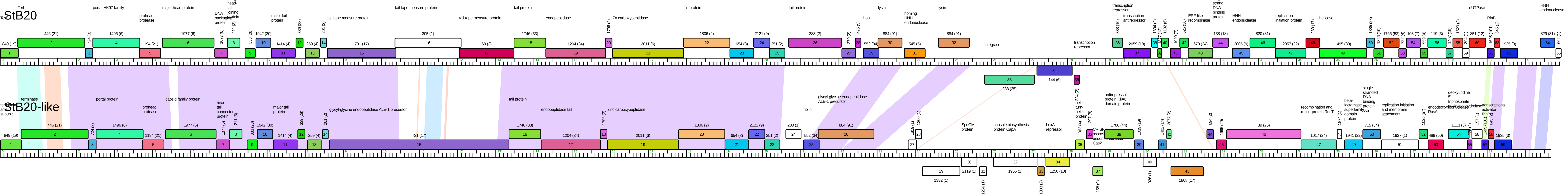

StB20
StB20-like
TerL
portal HK97 family
prohead protease
major head protein
major tail protein
tail tape measure protein
tail protein
endopeptidase
Zn carboxypeptidase
holin
lysin
homing HNH endonuclease
integrase
transcription repressor
transcription antirepressor
ERF-like recombinase
HNH endonuclease
replication initiation protein
helicase
dUTPase
HNH endonuclease
terminase
portal protein
capsid family protein
head-tail connector protein
glycyl-glycine endopeptidase ALE-1 precursor
endopeptidase tail
zinc carboxypeptidase
SpoOM protein
capsule biosynthesis protein CapA
LexA repressor
antirepressor protein KilAC domain protein
recombination and repair protein RecT
single-stranded DNA-binding protein
replication initiation and membrane attachment
endodeoxyribonuclease RusA
deoxyuridine 5'-triphosphate nucleotidohydrolase
transcriptional activator RinB
