# Supplementary material for: Staphylococci phages display vast genomic diversity and evolutionary relationships
Source: BMC Genomics. 2019 May 9;20:357. doi: 10.1186/s12864-019-5647-8 (PMC6507118; doi:10.1186/s12864-019-5647-8)
Supplement: Supplementary file 30 — Whole-genome map of subcluster C6 phages. Represented as mentioned above. (PDF 76 kb) [file 12864_2019_5647_MOESM30_ESM.pdf]

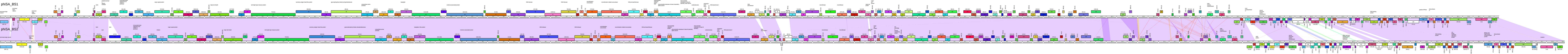
phiSA_BS1
phiSA_BS2
